# Fatty Acid Synthase Cooperates with Glyoxalase 1 to Protect against Sugar Toxicity

**DOI:** 10.1371/journal.pgen.1004995

**Published:** 2015-02-18

**Authors:** Damien Garrido, Thomas Rubin, Mickael Poidevin, Brigitte Maroni, Arnaud Le Rouzic, Jean-Philippe Parvy, Jacques Montagne

**Affiliations:** 1 Université Paris-Sud 11, Orsay, France; 2 CNRS, Centre de Génétique Moléculaire, UPR 3404, Gif-sur-Yvette, France; 3 CNRS, Laboratorie Evolution Genome et Speciation, UPR 9034, Gif-sur-Yvette, France; Max Planck Institute for Biophysical Chemistry, GERMANY

## Abstract

Fatty acid (FA) metabolism is deregulated in several human diseases including metabolic syndrome, type 2 diabetes and cancers. Therefore, FA-metabolic enzymes are potential targets for drug therapy, although the consequence of these treatments must be precisely evaluated at the organismal and cellular levels. In healthy organism, synthesis of triacylglycerols (TAGs)—composed of three FA units esterified to a glycerol backbone—is increased in response to dietary sugar. Saturation in the storage and synthesis capacity of TAGs is associated with type 2 diabetes progression. Sugar toxicity likely depends on advanced-glycation-end-products (AGEs) that form through covalent bounding between amine groups and carbonyl groups of sugar or their derivatives α-oxoaldehydes. Methylglyoxal (MG) is a highly reactive α-oxoaldehyde that is derived from glycolysis through a non-enzymatic reaction. Glyoxalase 1 (Glo1) works to neutralize MG, reducing its deleterious effects. Here, we have used the power of *Drosophila* genetics to generate *Fatty acid synthase* (*FASN*) mutants, allowing us to investigate the consequence of this deficiency upon sugar-supplemented diets. We found that *FASN* mutants are lethal but can be rescued by an appropriate lipid diet. Rescued animals do not exhibit insulin resistance, are dramatically sensitive to dietary sugar and accumulate AGEs. We show that FASN and Glo1 cooperate at systemic and cell-autonomous levels to protect against sugar toxicity. We observed that the size of *FASN* mutant cells decreases as dietary sucrose increases. Genetic interactions at the cell-autonomous level, where glycolytic enzymes or Glo1 were manipulated in *FASN* mutant cells, revealed that this sugar-dependent size reduction is a direct consequence of MG-derived-AGE accumulation. In summary, our findings indicate that FASN is dispensable for cell growth if extracellular lipids are available. In contrast, FA-synthesis appears to be required to limit a cell-autonomous accumulation of MG-derived-AGEs, supporting the notion that MG is the most deleterious α-oxoaldehyde at the intracellular level.

## Author Summary

Consumption of sugar and lipid (fat) enriched food increases the risk of developing metabolic diseases and cancers. However, lipids are essential molecules for life, as they are the major components of cell membranes. Metabolism refers to biochemical reactions that transform nutrients into molecules required by an organism, although toxic by-products can also formed. Sugars or their derivatives are likely to induce toxic effects by forming stable conjugates with proteins. To neutralize their toxic potential, sugars are metabolized and stored as fat. Here, we have used the fruitfly model to investigate the consequences of lipogenesis deficiency upon ingestion of sugar-enriched diets. We show that lipogenesis deficient animals are dramatically sensitive to dietary sugar. Further, we have identified the sugar by-product responsible for intracellular toxicity, in the context of lipogenesis inhibition. Our study reveals that inhibiting lipogenesis does not disrupt cellular growth if extracellular lipids are available. In contrast lipogenesis inhibition may have deleterious consequences due to accumulation of toxic by-products. The efficacy of lipogenic inhibitors in fighting cancers and metabolic diseases is currently under investigation. Therefore, to evaluate the clinical benefit of these inhibitors, accumulation of the toxic molecules should be monitored in both sick and healthy cells.

## Introduction

Deregulation of metabolism occurs in several pandemic human diseases whose incidence has dramatically increased due to changes in lifestyle and extended lifespan. These disorders include metabolic syndrome and type 2 diabetes (T2D) that are typified by insulin resistance and elevated levels of glucose and triacylglycerols (TAGs) in the plasma [1,2]. However, insulin resistance does not directly depend on an increase in TAG levels, but is rather a consequence of diacylglycerol and/or ceramides accumulation [1,3,4], whose levels increase as adipose tissue reaches a saturating point [5,6]. Cancer cells also exhibit metabolic perturbations characterized in part by a dramatic increase in glycolysis and fatty acid (FA) synthesis [7,8]. These changes emphasize direct links between sugar catabolism and FA synthesis.

Recent studies support the notion that glycation of proteins, DNA and/or phospholipids is likely to be responsible for the toxic effects induced by excess sugar [9,10]. The resulting compounds, advanced-glycation-end-products (AGEs), maybe responsible for vascular complication, nephropathy and retinal degeneration in T2D patients [11,12]. Glycation is a spontaneous reaction that occurs between an amine group and a carbonyl group of sugars or α-oxoaldehydes [13]. The latter include methylglyoxal (MG) that largely derives from spontaneous oxidation of the glycolytic intermediates dihydroxyacetone-phosphate (DHAP) and glyceraldhehyde-3-phosphate (G3P) [14]. The glyoxalase system [15], an enzymatic system composed of glyoxalase 1 (Glo1) and glyoxalase 2, maintains tolerable levels of MG.

In healthy organisms, circulating glucose is taken up by cells and is used to produce energy through glycolysis and the citric acid cycle. In postprandial condition, dietary glucose is used to synthesize glycogen in the liver and muscles. Excess glucose is also used for FA synthesis in hepatocytes and adipocytes. Synthesis of FA first requires carboxylation of acetyl-CoA to malonyl-CoA by the enzyme ACC (Acetyl-CoA carboxylase) [16]. Next, the Fatty acid synthase (FASN according to the current mammalian nomenclature) sequentially incorporates several malonyl-CoA molecules onto an acetyl-CoA primer to form a long chain FA (LCFA) [17].

*Drosophila* genetics has proven a powerful model system to investigate metabolic regulation at the level of the organism [18,19,20]. We previously demonstrated that in larvae, ACC is cell-autonomously required for the synthesis and storage of TAGs in the fat body (FB) [21], an insect organ with hepatic and adipose functions. We also provided evidence that within the oenocytes—abdominal cells with a hepatic-like function [22]—ACC is required to maintain the watertightness of the tracheal system [21].

Here, we have focused on the *Drosophila FASN* orthologs, of which only one (*FASN* ^*CG3523*^) is ubiquitously expressed. By directing inducible RNA-interfering (RNAi) to *FASN* ^*CG3523*^ and *glycogen synthase* (*GlyS*), we observed that the larval FB synthesizes both TAGs and glycogen. Next, we observed that expression of *FASN* ^*CG3523*^ is induced by dietary sugar and that *FASN* ^*CG3523*^ deficient animals are extremely sensitive to moderate increases in dietary sugar. Furthermore, we provide evidence that the activity of FASN and Glo1 cooperate both at the organismal and cellular level to protect against sugar toxicity.

## Results

### *FASN* genes in *Drosophila*

To investigate the physiological consequences of FA synthesis defect in *Drosophila*, we focused on the ortholog of the anabolic enzyme FASN, encoded by three distinct genes (*FASN* ^*CG3523*^, *FASN* ^*CG3524*^, *FASN* ^*CG17374*^) [21]. Previous reports show that in larval tissues, *FASN* ^*CG3523*^ is ubiquitously expressed, while *FASN* ^*CG3524*^ and *FASN* ^*CG17374*^ are mostly expressed in the carcass, which is comprised of epidermal cells, oenocytes and skeletal muscles [23]. To corroborate these findings, transcript levels of the three *FASN* genes were monitored using quantitative-PCR (RT-Q-PCR) in third stage larvae (L3) separated in two fractions, the internal organs, which can be easily removed and the leftover carcass. Consistently, *FASN* ^*CG3523*^ transcripts were detected at high levels in both fractions, whereas *FASN* ^*CG3524*^ and *FASN* ^*CG17374*^ transcripts were detected at high levels in the carcass, but minimally in the internal organs (S1A Fig.).

To determine whether these enzymes are essential, we made use of the binary Gal4/UAS system to direct specific RNAi to each *FASN* gene and to the *ACC* orthologue [21]. Ubiquitous knockdown of these genes caused lethality at late embryogenesis for *ACC*, at L1 stage for *FASN* ^*CG3523*^, at the L2 stage for *FASN* ^*CG17374*^, while no phenotype was observed for *FASN* ^*CG3524*^ (Table 1). The lethality at the L2 stage observed in *FASN* ^*CG17374*^ *-RNAi* knockdown resembled the phenotype previously observed when inducing this RNAi using an oenocyte specific driver [21], typified by a defect in the watertightness of the tracheal system (S1B–S1C Fig.). Therefore, we have used a *svp-gal80* transgene to inhibit Gal4 in the oenocytes [22]. Driving *FASN* ^*CG17374*^ *-RNAi* in the entire animal, except in the oenocytes, resulted in a total rescue of the lethal phenotype (Table 1 and S1D Fig.), indicating that *FASN* ^*CG17374*^ does not serve an essential function in other tissues. To get further insights into the organ-specific function of these enzymes, we used the *Cg-gal4* and *Mef2-gal4* drivers, which are specific to the FB and the muscles, respectively. When induced in either tissue, knockdown to any of either gene did not affect viability (Table 1). Nonetheless, muscle knockdown of *ACC* or *FASN* ^*CG3523*^, but not of *FASN* ^*CG3524*^ or *FASN* ^*CG17374*^, led to a motility defect in adult flies (Table 1). Taken together, these findings indicate that the synthesis of LCFA is not essential in either the FB or the muscles. However, consistent with previous studies reporting that muscle-specific knockdown of *ACC* affects body homeostasis and motility of adult flies [24,25], our findings indicate that FA synthesis plays an important role in muscle development and/or activity.

**Table 1:** Genotypic analysis of *ACC* and of the 3 *FASN* genes using ubiquitous and tissues-targeted knockdown.

Driver	*da-gal4*	*Cg-gal4*	*Mef2-gal4*
	Ubiquitous	Fat body	Muscles
*ACC-Ri*	† embryo	viable	Motility default
*FASN^CG3523^-Ri*	† L1	viable	Motility default
*FASN^CG3524^-Ri*	viable	viable	No defect
*FASN^CG17374^-Ri*	† @4-d; L2	viable	No defect
*FASN^CG17374^-Ri;svp-gal80*	viable	ND	ND

Column 1 lists the *UAS-RNAi* induced and the *svp-gal80* transgene when used. Column 2, 3 and 4 indicate the phenotype obtained using the ubiquitous-, FB- and muscle-specific drivers, respectively. Lethality (†) may occur at embryogenesis (embryo), at L1 or L2 stages, 4 days after egg deposit (@4-d). Flies were considered to have a motility defect if they failed to climb up the wall of the feeding tube, as flies usually do. Each test was repeated at least 3 times.

### *FASN* ^*CG3523*^ knockdown affects TAG and Glycogen levels

We previously reported that FB-knockdown of *ACC* results in a decrease in total TAG levels [21]. To determine which of the three FASN members is necessary for LCFA synthesis in the FB, RNAi to each *FASN* gene was induced using the FB-specific driver. Consistent with the finding that *FASN* ^*CG3523*^ is the only *FASN* gene expressed in internal organs (S1A Fig.), total TAG levels were dramatically reduced in *FASN* ^*CG3523*^ but not in *FASN* ^*CG17374*^ and *FASN* ^*CG3524*^ knockdowns (S2A Fig.).

We previously observed that in *Cg>ACC-RNAi* (*Cg-gal4* directing *ACC-RNAi*) animals the drop in whole larvae TAG levels was accompanied by an increase in glycogen storage [21]. Thus, to investigate the physiological relationship between TAG and glycogen storage, RNAi transgenes to either *ACC* or *FASN* ^*CG3523*^ was combined with an RNAi transgene to the gene encoding the unique *Drosophila* GlyS. Single or dual knockdowns were induced in either the FB, the muscles or in both tissues. Total amounts of TAG, glycogen, trehalose, glucose and protein were measured in 0–5h prepupae, as this is a convenient phase to stage the animals after the feeding period. Prepupal weighing revealed that animals expressing *GlyS-RNAi* in combination with either *ACC-RNAi* or *FASN* ^*CG3523*^ *-RNAi* in both the muscles and the FB exhibited the most prominent reduction in body weight (S2B Fig. and S1 Table). Total TAG levels decreased dramatically when either *ACC-RNAi* or *FASN* ^*CG3523*^ *-RNAi* were induced in the FB but not in the muscles (S2C Fig.); this decrease was roughly similar when either RNAi were induced in both tissues (S2C Fig.). Furthermore, TAG levels measured in control, *ACC-RNAi-* or *FASN* ^*CG3523*^ *-RNAi*-expressing animals were not markedly modified by the expression of the *GlyS-RNAi* (S2C Fig.). Total glycogen levels decreased when *GlyS-RNAi* was expressed in either the FB or the muscles, and decreased further when *GlyS-RNAi* was expressed in both tissues (S2D Fig.), indicating that in prepupae both organs contribute to glycogen storage. This finding contrasts with a previous study reporting that in larvae, glycogen can be detected in skeletal muscles only [26]. Therefore, since glycogen is unlikely to be transported between organs, it is conceivable that FB glycogen synthesis mostly occurs at late larval stage. Unexpectedly, driving either *ACC-RNAi* or *FASN* ^*CG3523*^ *-RNAi* in the muscles provoked a moderate decrease in glycogen levels (S2D Fig.). This may be a consequence of muscle dysfunction linked to the above mentioned motility defect (Table 1). Importantly, FB-knockdown to *ACC* or *FASN* ^*CG3523*^ provoked a very strong increase in total glycogen levels that is not observed when co-expressing *GlyS-RNAi* (S2D Fig.), indicating that this extra-glycogen is synthesized inside the FB. Furthermore, we observed that trehalose levels (S2E Fig.) in part mirrored the variations observed with in glycogen (S2D Fig.), as shown by a strong correlation (S2F Fig.). Since energy stores are mobilized during metamorphosis, the decrease in trehalose levels might be a direct consequence of reduced glycogen breakdown. However, when *ACC-RNAi* or *FASN* ^*CG3523*^ *-RNAi* was expressed in the FB, glycogen but not trehalose levels increased dramatically (S2D–S2F Fig.), suggesting that trehalose levels cannot be increased in the prepupae. Finally, neither glucose (S2G Fig.) nor protein (S2H Fig.) levels exhibited severe perturbation in any of the tested genotypes. Taken together these results indicate that at the end of larval life, glycogen accumulates in both the muscles and the FB, whereas TAGs accumulate mainly in the FB. Further, a reduction in TAG storage can, in part, be compensated for by an increase in glycogen storage.

### Direct links between FA synthesis and sugar metabolism

**Fig 1:**
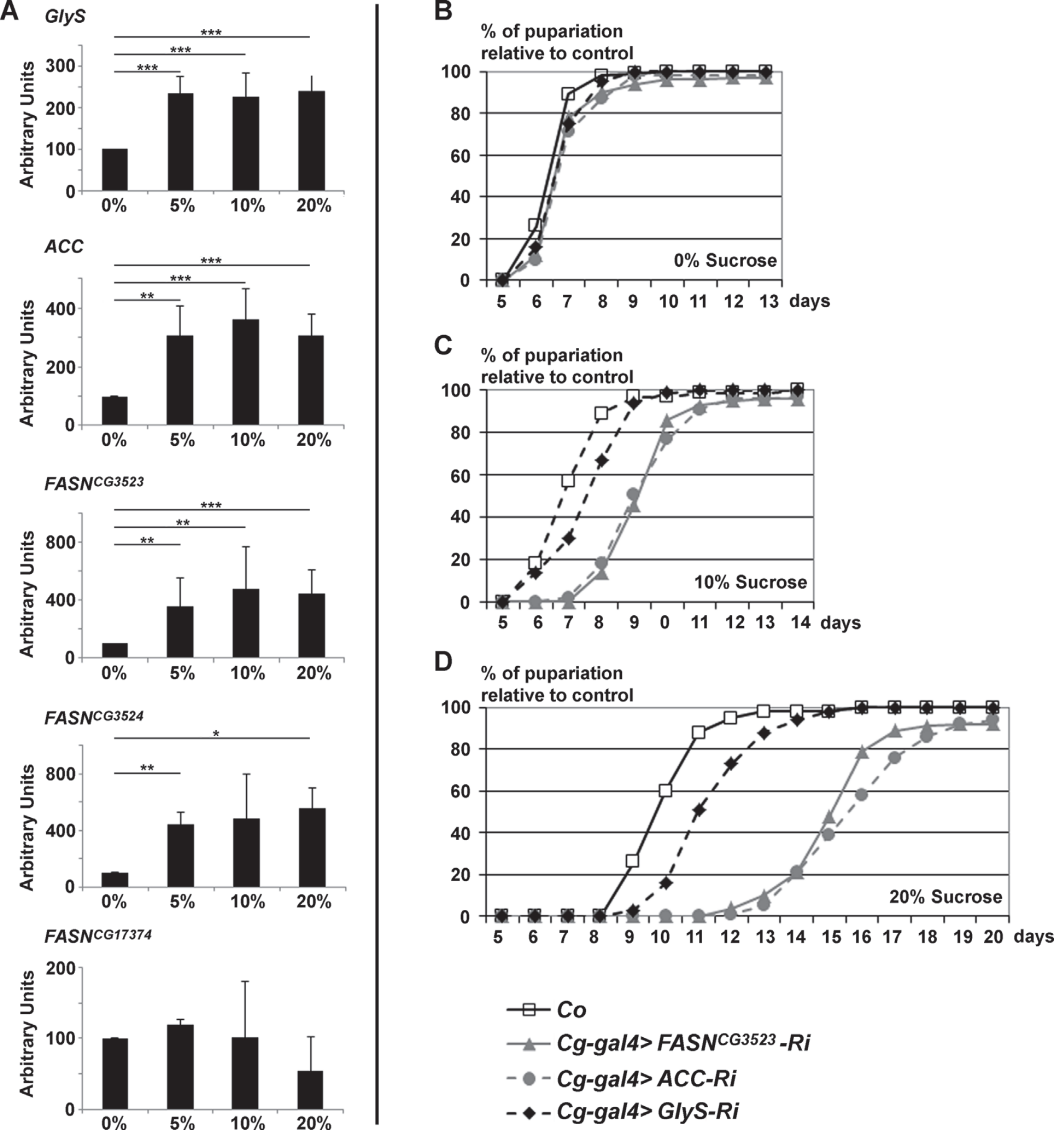
*GlyS*, *ACC and FASN* ^*CG3523*^ expression is induced by and protects from dietary sugar. (A) RT-Q-PCR (means calculated from 3 samples of 10 feeding L3 larvae) to *GlyS*, *ACC*, *FASN* ^*CG3523*^, *FASN* ^*CG3524*^ and *FASN* ^*CG17374*^ in response to increasing concentration of dietary sucrose (0%, 5%, 10%, 20%). (B-D) Developmental delay was measured at puparium formation of larvae fed a LCD (B), a 10%-SSD (C) or a 20%-SSD (D). The *Cg-gal4* driver was used to direct RNAi to *GlyS*, *ACC* or *FASN* ^*CG3523*^ within the FB. The *Cg-gal4* was combined with a *UAS-Dcr2* transgene to strengthen the RNAi effect. Controls (Co) were progeny resulting from the cross between *Cg-gal4* females and *w* ^*-*^ balanced males. In (B-D), each curve represents at least 300 animals; experiment repeated twice.

Considering that the synthesis of glycogen and TAG constitutes a metabolic mechanism to safely store high quantities of glucose, we hypothesized that the anabolic enzymes FASN, ACC and GlyS, are induced by dietary sugar. Therefore larvae were fed a low carbohydrate diet (LCD) or a sucrose-supplemented diet (SSD). Using RT-Q-PCR, the expression of *ACC*, *GlyS* and the three *FASN* genes was monitored in larvae fed on 0% (LCD), 5%-, 10%- and 20%-SSDs (S2 Table). *FASN* ^*CG17374*^ expression was insensitive to increases in dietary sugar, while the expression of all the other genes was enhanced by sucrose (Fig. 1A). This response was observed following a 5%-SSD but was not further enhanced by 10%- and 20%-SSD, indicating that a moderate increase in dietary sugar elicits an adaptive metabolic response.

Next, we wondered whether the synthesis of FA may protect against excess dietary sugar. Considering that the FB is the main storage organ, RNAi to *ACC*, *FASN* ^*CG3523*^, or *GlyS* were induced with the *Cg-gal4* driver and the duration of larval development was monitored by following the onset of metamorphosis. When fed LCD, no developmental delay was observed in control, *Cg>ACC-RNAi*, *Cg>FASN* ^*CG3523*^ *-RNAi* or *Cg>GlyS-RNAi* larvae (Fig. 1B). In contrast, when fed a 10%-SSD, the onset of metamorphosis was delayed by roughly two days in *Cg>ACC-RNAi* and *Cg>FASN* ^*CG3523*^ *-RNAi* larvae, while *Cg>GlyS-RNAi* larvae were only slightly delayed (Fig. 1C and lines 1–3, S3 Table). The effect was enhanced for larvae fed a 20%-SSD. Control larvae exhibited a 3-day delay, *Cg>GlyS-RNAi* larvae exhibited a 4-day delay, whereas *Cg>ACC-RNAi* and *Cg>FASN* ^*CG3523*^ *-RNAi* larvae exhibited approximately an 8-day developmental delay (Fig. 1D and lines 4–6, S3 Table). Together, these findings indicate that FA synthesis is a crucial metabolic pathway, which buffers the developmental defects induced by excess dietary sugar.

### Generation of *FASN* mutants

**Fig 2:**
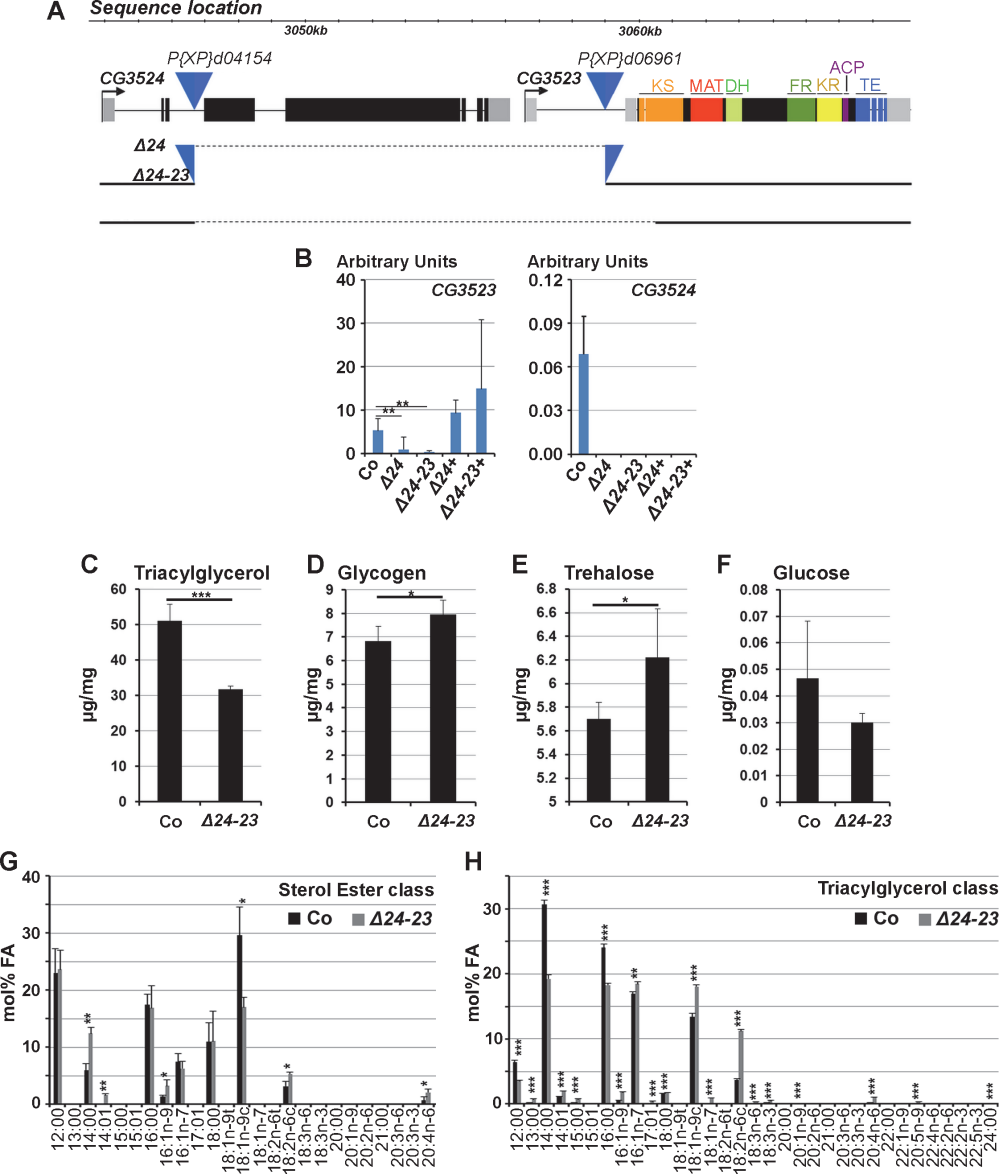
Characterization of *FASN* mutants. (A) Locus of the *FASN* ^*CG3523*^, *FASN* ^*CG3524*^ genes. The blue triangles show the two P-element insertions used to generate the *FASN* ^*Δ24*^ deficiency. The dotted lines indicate the genomic sequences removed in *FASN* ^*Δ24*^ and *FASN* ^*Δ24-23*^ mutants. The ketoacyl synthase (KS), malonyl acyl transferase (MAT), dehydratase (DH), enoyl reductase (ER) ketoreductase (KR), acyl carrier protein (ACP) and thioesterase (TE) domains of FASN^CG3523^ are indicated. (B) RT-Q-PCR (means calculated from 3 samples of 10 feeding L3 larvae) to *FASN* ^*CG3523*^ (left) and *FASN* ^*CG3524*^ (right) in *w* ^*-*^ control (Co), *FASN* ^*Δ24*^ (*Δ24*) and *FASN* ^*Δ24-23*^ (*Δ24-23*) mutants rescued by dietary lipids or the *UAS-FASN* ^*CG3523*^ transgene (*Δ24+* and *Δ24-23+*). (C-F) Concentration of TAGs (C), glycogen (D), trehalose (E) and glucose (F) in *w* ^*-*^ control (Co) or *FASN* ^*Δ24-23*^ (*Δ24-23*) mutant prepupae raised on the rescuing lipid media. (G-H) FA profiles of the sterol esters (G) and TAGs (H) classes from either *w* ^*-*^ control (Co) or *FASN* ^*Δ24-23*^ (*Δ24-23*) mutant prepupae raised on a beySD. Fatty acid species [71] are indicated at the bottom of each panel. TAGs values are means calculated from 5 samples of 150 mg 0–5h prepupae; glucose, trehalose and glycogen values are means calculated from 4 samples of 500 mg 0–5h prepupae. Fatty acid profiles represent means calculated from 3 samples of 100 mg 0–5h prepupae.

To gain further insights into the physiological requirements of LCFA synthesis we generated *FASN* mutants. As shown above, *FASN* ^*CG3523*^ is an essential gene ubiquitously expressed, whereas *FASN* ^*CG17374*^ sustains the synthesis of an essential FA only within the oenocytes (Table 1 and S1C–S1D Fig.). *FASN* ^*CG3524*^ is not essential (Table 1) and may be redundant with *FASN* ^*CG3523*^, as these two genes are in tandem on the second chromosome (Fig. 2A) and both are induced by dietary sugar (Fig. 1A). Therefore, we took advantage of two FRT-containing P-elements, located within the *FASN* ^*CG3524*^ and the *FASN* ^*CG3523*^ genes (Fig. 2A). Flipase recombination between the FRT sequences of these two P-elements resulted in a complete deletion of *FASN* ^*CG3524*^, hereafter referred to as *FASN* ^*Δ24*^. The resulting chimeric P-element links the 5’ region of *FASN* ^*CG3524*^ to most of the *FASN* ^*CG3523*^ genomic sequences (Fig. 2A). To generate a null *FASN* ^*CG3523*^ mutant, we performed a remobilization of the chimeric P-element and looked for imprecise excisions that remove part of the *FASN* ^*CG3523*^ gene. 22 excisions were recovered, one of them (hereafter referred to as *FASN* ^*Δ24-23*^) removed 1200-bp of the *FASN* ^*CG3523*^ gene (Fig. 2A), including the first methionine codon and the sequence coding half of the β-ketoacyl synthase (KS) domain [17].

Both *FASN* ^*Δ24*^ and *FASN* ^*Δ24-23*^ are lethal at the L1 larval stage. RT-Q-PCR analysis showed that *FASN* ^*CG3524*^ expression could not be detected in either mutants fed a lipid-supplemented diet (Fig. 2B and see below). In addition, *FASN* ^*CG3523*^ transcript levels were severely reduced in homozygous *FASN* ^*Δ24*^ larvae and barely detectable in homozygous *FASN* ^*Δ24-23*^ larvae (Fig. 2B). Therefore, both mutations delete *FASN* ^*CG3524*^, however, *FASN* ^*Δ24*^ appears to be a hypomorphic mutant and *FASN* ^*Δ24-*^23 a null mutant for *FASN* ^*CG3523*^.

To ascertain that the L1 lethality observed in both mutants was solely due to *FASN* deficiency, rescue experiments were performed, using UAS lines expressing either *FASN* ^*CG3524*^ or *FASN* ^*CG3523*^ cDNA. Ubiquitous overexpression revealed that *FASN* ^*CG3524*^ cDNA could partially rescue the lethality of *FASN* ^*Δ24-23*^ mutants to the pupal stage, although none emerged as adults (S4 Table). In contrast, ubiquitous overexpression of *FASN* ^*CG3523*^ cDNA did not rescue the lethal phenotype in either *FASN* mutants and induced embryonic lethality when driven with any of the ubiquitous *gal4*-lines tested (S4 Table). However, one of the *UAS-FASN* ^*CG3523*^ lines was able to partially rescue the lethal phenotype to pupal or adult stages in both mutants in the absence of *gal4* drivers (S4 Table). Consistently, RT-Q-PCR analysis revealed that *FASN* ^*CG3523*^ but not *FASN* ^*CG3524*^ transcripts were detected at high levels in both *FASN* mutant rescued animals (Fig. 2B), indicating that an endogenous promoter could drive the expression of this *UAS-FASN* ^*CG3523*^ transgene. These findings show that both *FASN* ^*Δ24*^ and *FASN* ^*Δ24-23*^ are *bona fide* mutants and suggest that FASN^CG3523^ protein levels should be maintained within a precise window of expression.

### Rescue of *FASN* mutants with dietary lipids

To determine whether the lethal phenotype could be rescued by dietary lipids, a LCD was supplemented with lipids (S2 and S5 Tables). Interestingly, supplementing a LCD with soy lipids could in part rescue the lethality of the hypomorph *FASN* ^*Δ24*^ mutant to pupal or adult stages (S5 and see below) but not the lethality of the null *FASN* ^*Δ24-23*^ mutant (S5 Table). We therefore, supplemented a LCD with various dietary lipids, including oils, margarine, butter and egg yolk alone or in combination. In isolation, none of the dietary lipids could rescue lethality of *FASN* ^*Δ24-23*^ mutants, although a few larvae grew and developed to the L2 or L3 stages (S5 Table). In contrast, a LCD supplemented with butter and egg yolk (beySD) (S2 Table) could partially rescue lethality of both *FASN* ^*Δ24*^ and *FASN* ^*Δ24-23*^ mutants (S5 Table). To evaluate the metabolic consequences of the *FASN* deletion, TAG, glycogen, trehalose and glucose levels were measured in the *FASN* ^*Δ24-23*^ mutant and control prepupae fed a beySD. *FASN* ^*Δ24-23*^ prepupae exhibited a net decrease in TAG levels (Fig. 2C) associated with a moderate increase in glycogen and trehalose levels (Fig. 2D-E), whereas glucose levels were not significantly modified (Fig. 2F). Then, we performed a detailed analysis of FA composition of the TAGs, the sterol esters, and the various phospholipid classes. This analysis revealed that the relative FA content of the various phospholipids was not significantly modified (S3A–S3F Fig.). In each phospholipid class, palmitic acid (16:00) was always the most abundant FA component, although palmitoleic (16:01), stearic (18:00) oleic (18:01) and linoleic (18:02) acids were also highly represented. In contrast, the relative FA content in the sterol ester and TAG classes significantly varied in *FASN* ^*Δ24-23*^ mutants *versus* controls (Fig. 2G-H). For the sterol ester class, oleic acid was less abundant in the mutants than in the control; however, this deficit was compensated for with higher levels of myristic (14:00), myristoleic (14:01), palmitoleic, linoleic (18:02) and arachidonic (20:04) acids (Fig. 2G). For the TAG class, control prepupae contained a higher proportion of saturated lauric (12:00), myrictic and palmitic acids, whereas mutants contained a higher proportion of unsaturated myristoleic, palmitoleic, oleic, linoleic and arachidonic acids (Fig. 2H). Together, these findings suggest that dietary lipids provide phospholipid precursors in sufficient amounts to compensate for the loss of FASN. Further, the difference in the FA composition of the TAG class in mutant *versus* control animals suggests that the structure of the TAGs is not critical.

### Moderate sucrose supplementation is dramatic for FASN deficient animals

**Fig 3:**
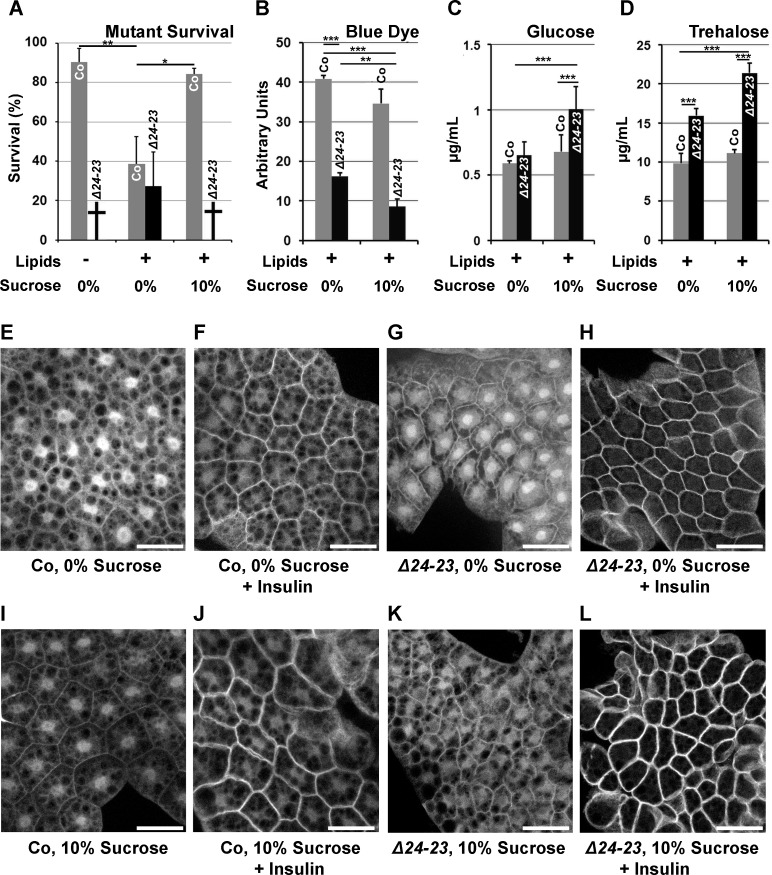
Sugar sensitivity of *FASN* null mutants. (A) Survival until metamorphosis or early larval lethality (**†**) of *w* ^*-*^ control (Co) and *FASN* ^*Δ24-23*^ mutant (*Δ24-23*) animals fed either a LCD (Lipids -) or a beySD (Lipids +) with (10%) or without (0%) additional sugar. For each condition, the mean of survival rate was calculated by assessing the number of newly hatched larvae reaching metamorphosis (groups of 100 L1 larvae placed in 5 separate tubes). (B-D) Metabolic measurements from *w* ^*-*^ control (Co) or *FASN* ^*Δ24-23*^ mutant (*Δ24-23*) larvae that were fed a beySD until L2/L3 transition and then transferred onto fresh media (0%) or media containing additional sucrose (10%) for 24h. (B) Blue dye accumulation in 24h old L3 larvae after feeding on a tinted media for 1h (means calculated from 3 samples of 10 feeding L3 larvae); experiments repeated 3 times. (C-D) Circulating glucose (C) and trehalose (D) levels from bled larvae (means calculated from 4 samples of 20 to 30 bled L3 larvae); experiment repeated 3 times. (E-L) Membrane localization of tGPH in FB explants incubated with (F,H,J,L) or without (E,G,I,K) insulin (0,5 μM) (for quantification of tGPH intensity see S4B Fig.). FB explants were dissected from *w* ^*-*^ control (E,F,I,J) or *FASN* ^*Δ24-23*^ (G,H,K,L) L3 larvae that were fed a beySD (E-H) or the same media supplemented with 10% sucrose (I-L) for 24h. For each genotype, at least 10 larvae were dissected; experiment repeated twice. Scale bars: 20μm.

Next, we investigated sucrose sensitivity in *FASN* mutant. Importantly, about 40% of the *FASN* ^*Δ24*^ mutants fed a soy-lipid supplemented diet and of the *FASN* ^*Δ24-23*^ mutant fed a beySD underwent metamorphosis onset (S4A Fig. and Fig. 3A). As shown by standard deviation values the percentages of rescue was highly variable. Nonetheless, addition of 10% sucrose to either lipid supplemented diet, resulted in a total lethality at L1 stage for both *FASN* mutants (S4A Fig. and Fig. 3A). These findings indicate that individuals that are unable to synthesized FAs are extremely sensitive to moderate increases in dietary sucrose. Moreover, less than half of the control larvae were able to pupariate when fed a lipid supplemented diet (S4A Fig. and Fig. 3A). The lipotoxicity was markedly suppressed when beySD was supplemented with 10% sucrose (Fig. 3A), possibly due to a reduction in the feeding rate (see below).

Since metabolic analysis is easier to perform on late rather than early larvae—which are very small—, a diet-shift protocol was established. *FASN* ^*Δ24-23*^ mutant and control larvae were fed a beySD until the L2/L3 transition, transferred onto the same feeding media with or without 10% sucrose supplementation and left to develop 24h or 40h. First, to evaluate the feeding rate, larvae were transferred onto fresh media stained with brilliant blue FCF dye, and absorption of stained food was evaluated from whole larval extracts after one hour. Colorimetric measurement revealed that *FASN* ^*Δ24-23*^ mutants contained much less food in their gut than control animals (Fig. 3B), and that sucrose supplementation also reduced the stained food content in both *FASN* ^*Δ24-23*^ and control larvae (Fig. 3B). The lower gut content suggests that food uptake was reduced, although we could not exclude an increase in stool elimination. Next, levels of circulating sugars in larval hemolymph were measured. Interestingly, neither glucose nor trehalose levels increased in control larvae fed a 10%-sucrose supplemented beySD (Fig. 3C-D), suggesting that this feeding protocol does not induce a diabetic-like phenotype. Nonetheless, *FASN* ^*Δ24-23*^ mutants fed a beySD exhibited a moderate increase in trehalose levels (Fig. 3D), while glucose levels remained unchanged (Fig. 3C). In contrast, after 24h of feeding on a 10%-sucrose supplemented beySD, both glucose and trehalose levels were strongly increased (Fig. 3C-D). Considering that increases in levels of circulating sugar is a hallmark of diabetes [27], the insulin response was evaluated in the FB of larvae expressing a tGPH reporter [28]. FBs were dissected from larvae fed a beySD with or without a 10%-sucrose supplement, and membrane translocation of tGPH was analyzed after 20 mn incubation with or without insulin. When grown on either feeding media, both control and mutant FBs were highly responsive to insulin stimulation (Fig. 3E-L and S4B Fig.) indicating that neither the *FASN* ^*Δ24-23*^ mutant nor control larvae exhibit a T2D-like phenotype when fed a 10%-sucrose supplemented beySD. Importantly, the membrane-GFP fluorescence induced by insulin stimulation was much higher in *FASN* ^*Δ24-23*^ mutant than in control larvae (Fig. 3F,H,J,L and S4B Fig.), suggesting that the former were hypersensitive to insulin. Together, our findings indicate that *FASN* ^*Δ24-23*^ mutant animals are highly sensitive to dietary sugar but do not exhibit a T2D-like phenotype.

### FASN and Glo1 cooperation

**Fig 4:**
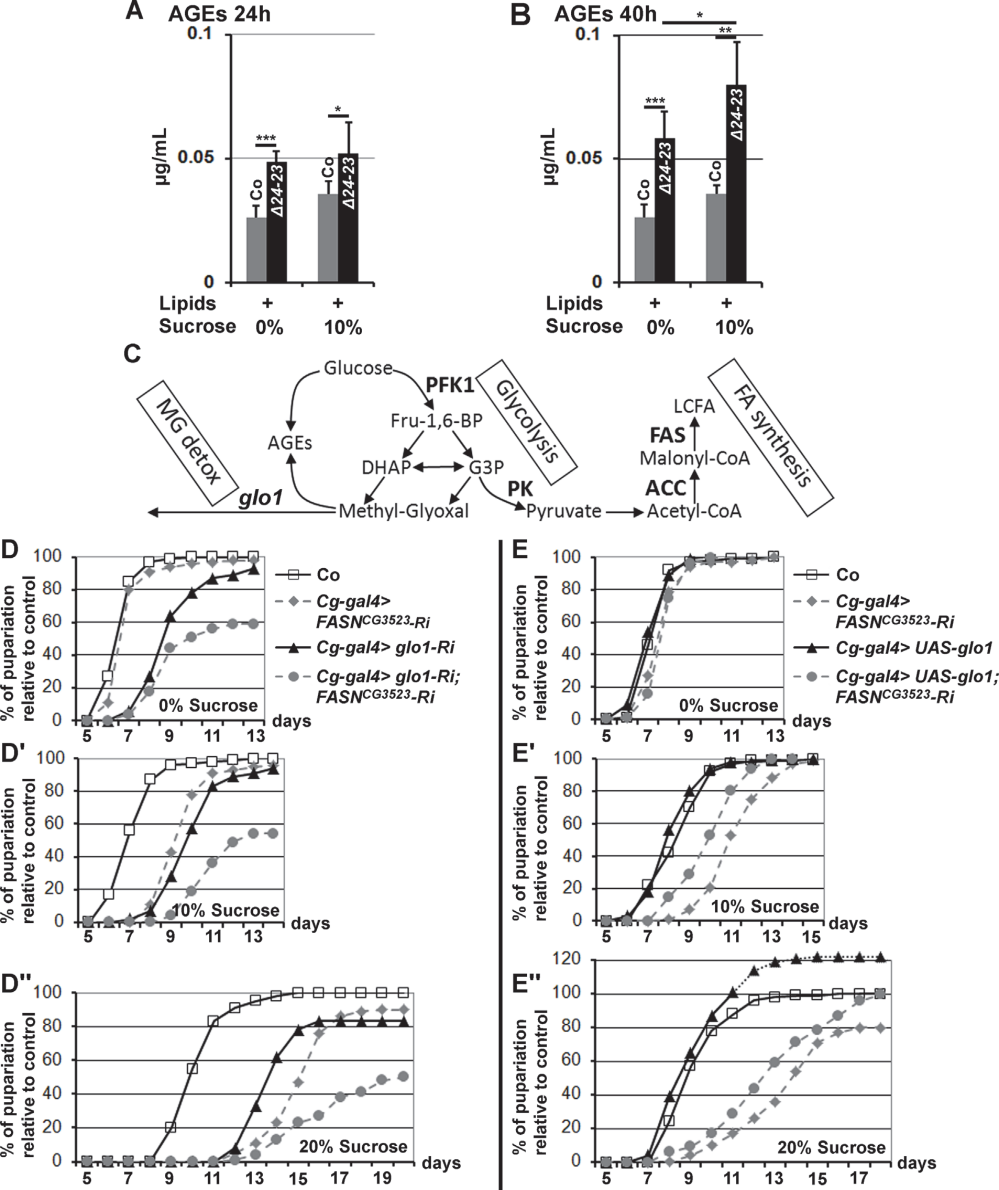
AGE metabolism and *FASN* deficiency. (A-B) AGE levels in *w* ^*-*^ control (Co) and *FASN* ^*Δ24-23*^ mutant (*Δ24-23*) L3 larvae raised for 24h (A) or 40h (B) (calculated from 5 samples of 10 L3 larvae); lipid and sucrose complements are indicated as in Fig. 3A-D; experiment repeated twice. (C) Metabolic links between glucose catabolism, FA synthesis, AGE formation and MG detoxification. Enzymes are indicated in bold characters. (D-D”,E-E”) Developmental delay measured at puparium formation of larvae fed a LCD (D,E), a 10%-SSD (D’,E’) or a 20%-SSD (D”,E”). (D-D”) The *Cg-gal4* driver was used to direct RNAi to *glo1*, *FASN* ^*CG3523*^ or both together within the FB. In (D-D”), each curve represents at least 300 animas; experiment repeated twice. (E-E”) The *Cg-gal4* driver was used to direct *FASN* ^*CG3523*^ *-RNAi*, *UAS-glo1*, or both together. Controls (Co) are progeny resulting from the cross between *Cg-gal4* females and *w* ^*-*^ balanced males. In (E-E”), each curve represents at least 700 animals.

Since an increase in AGEs is linked to high levels of circulating sugar in T2D patients [12], we compared the amounts of AGEs in whole control or *FASN* mutant larvae. In L3 larvae transferred onto fresh beySD for 24h, the amounts of AGEs were higher in *FASN* ^*Δ24-23*^ mutants than in controls. This was the case regardless or whether the beySD was supplemented with 10% sucrose (Fig. 4A). In older L3 larvae transferred on fresh beySD for 40h, the amounts of AGEs were strongly increased in *FASN* ^*Δ24-23*^ mutants compare to controls (Fig. 4B). In addition, exposure to 10%-sucrose supplemented beySD further increased AGE levels in *FASN* ^*Δ24-23*^ mutants (Fig. 4B), suggesting that FA synthesis constitutes a metabolic pathway to restrict AGE accumulation.

To further investigate the effects of dietary sucrose, we performed RNAi knockdown to two glycolytic enzymes encoded by single genes, Phosphofructokinase 1 (PFK1) and Pyruvate kinase (PK) that catalyze an early and the last glycolytic steps, respectively (Fig. 4C). FB-targeted knockdown to either PFK1 or PK did not result in a phenotypic defect in larvae fed a LCD, as developmental times did not differ markedly from controls (S5A Fig.). However these larvae were very sensitive to sucrose. When fed a 10%-SSD, both RNAi-knockdown larvae exhibited a significant developmental delay (S5B Fig. and lines 7–8, S3 Table). Moreover, when fed a 20%-SSD, the developmental delay was further increased for *Cg>PFK1-RNAi* larvae, whereas most of the *Cg>PK-RNAi* animals died during larval life (S5C Fig. and lines 9–10, S3 Table). The difference in sucrose sensitivity suggests either that *PK-RNAi* induces a more efficient knockdown than *PFK1-RNAi*, or that some glycolytic intermediates produced downstream of the enzymatic step catalyzed by PFK1 are extremely toxic.

Following the glycolytic step catalyzed by PFK1, an Aldolase cleaves fructose 1,6 bisphophate (Fru-1,6-BP) in the trioses phosphate, DHAP or G3P. Either metabolite leads to pyruvate, or to the highly reactive glycating α-oxoaldehyde MG via a non enzymatic reaction (Fig. 4C). We therefore used *UAS-RNAi* to the single *glo1* ortholog that encodes an MG neutralizing enzyme. *FASN* ^*CG3523*^ *-RNAi* and *glo1-RNAi* were induced independently or together in the FB and the duration of larval development was monitored. When fed a LCD, *glo1-RNAi* larvae exhibited a moderate developmental delay (Fig. 4D and line 11, S3 Table). This developmental delay was slightly prolonged when fed a SSD, although not to the same extent as *FASN* ^*CG3523*^ *-RNAi* larvae, which were much more sensitive to dietary sucrose (Fig. 4D-D” and lines 11,14,17, S3 Table). Furthermore, animals dually expressing *FASN* ^*CG3523*^ *-RNAi* and *glo1-RNAi* in their FB exhibited a high rate of larval lethality and a developmental delay that dramatically increased concurrently with sucrose concentration (Fig. 4D-D” and lines 12–13,15–16,18–19, S3 Table). Conversely, FB-overexpression of Glo1 was able to partially compensate for the developmental delay induced by an increase dietary sugar (Fig. 4E-E” and lines 20,22, S3 Table). FB-overexpression of Glo1 was also able to partially suppress the strong developmental delay of *FASN* ^*CG3523*^ *-RNAi* larvae grown on SSD (Fig. 4E-E” and lines 21,23, S3 Table). In each assay, the percentage of pupae is relative to the number of their *SM5-TM6B* siblings (see material and methods). Intriguingly, when fed a 20%-SSD, the ratio of *UAS-glo1* larvae relative to the number of their *SM5-TM6B* siblings was higher than the control ratio, reaching a maximum at roughly 120% (Fig. 4E”). Furthermore, we also observed that when testing homozygous *w* ^*-*^ control flies, the rate of larval lethality was significantly higher in 20%-SSD than in LCD or in 10%-SSD (S5D Fig.). This observation suggests that in the Glo1-overexpressing assay, a significant number of the *SM5-TM6B* siblings underwent lethality when fed a 20%-SSD and that Glo1 overexpression suppresses this lethality. In contrast in the control assay all the larvae underwent the same rate of lethality irrespective of the *SM5-TM6B* balancers. Together, these findings indicate that sucrose toxicity can be alleviated by overexpression of Glo1 and conversely, the deleterious effects are exacerbated when both FA synthesis and Glyoxalase activity are simultaneously dampened.

### Cell-autonomous sucrose toxicity

**Fig 5:**
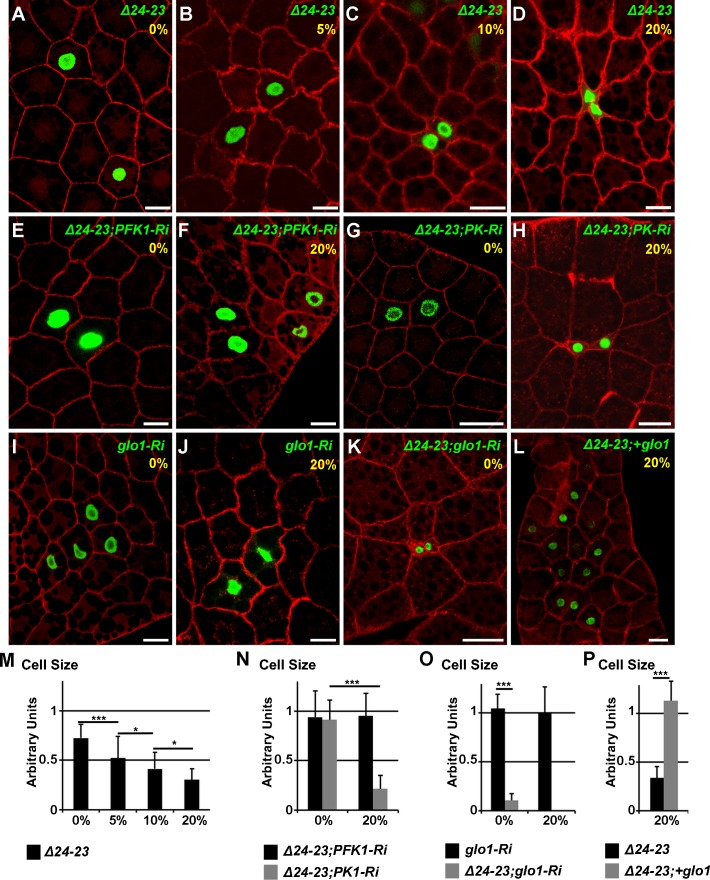
Cell-autonomous defect in *FASN* mutant FB cells. (A-L) Phalloidin staining of FB from feeding L3 larvae containing MARCM clones labeled with GFP. At the top right corner of each image, the genotype of the clonal cells and the percentage of sucrose supplementation are shown in green and yellow, respectively. *FASN* ^*Δ24-23*^ (*Δ24-23*), *PFK1-RNAi* (*PFK1-Ri*), *PK-RNAi* (*PK-Ri*), *glo1-RNAi* (*glo1-Ri*) and *UAS-glo1* (+*glo1*). Scale bars: 20μm. (M-P) Size ratio between at least ten clonal cells and the neighbouring control cells, as shown in A, B, C, D (M), E, F, G, H (N), I, J, K (O) and L (P). For each condition, at least 10 larvae were dissected; whilst searching for *FASN* ^*Δ24-23*^ clones expressing *glo1-RNAi* in larvae fed a 20%-SSD at least 40 animals were dissected.

To determine whether a lack of FA synthesis induces cell-autonomous defects, we generated flip-out recombination during embryogenesis and analyzed the resulting clones in the FB of feeding larvae at the end of the L3 stage. Interestingly, the size of *FASN* ^*CG3523*^ *-RNAi* cells was almost normal in larvae fed a LCD, but drastically reduced in larvae fed a 20%-SSD (S6A–S6B,S6M Fig.). A similar phenotype was observed for *PK-RNAi* flip-out cells (S6C–S6D,S6M Fig.), although the size reduction observed in larvae fed a 20%-SSD, varied a lot depending on the experiment, possibly because of variability in RNAi efficiency. In contrast, *PFK1-RNAi* flip-out cells were insensitive to dietary sucrose since cell size remained unchanged irrespective of sucrose supplementation (S6E–S6F,S6M Fig.). To perform genetic interactions at the cellular level, we generated MARCM clones either mutant (*FASN* ^*Δ24-23*^) or wild-type (*FASN* ^*+*^). Firstly, the sucrose sensitivity of *FASN* ^*Δ24-23*^ cells was evaluated in the FB of larvae raised on media containing increasing quantities of sucrose. For larvae fed a LCD, the size of *FASN* ^*Δ24-23*^ cells was slightly reduced compare to neighboring control cells (Fig. 5A,M). However, as the sucrose content in the diet increased, a concomitant reduction in the size of *FASN* ^*Δ24-23*^ cells was observed (Fig. 5B-D,M). This cell size reduction was not correlated with lipid content, as Nile red staining revealed that *FASN* ^*Δ24-23*^ cells were severely depleted in LDs, irrespective of sugar supplementation (S6G–S6I Fig.). Next, we generated *FASN* ^*Δ24-23*^ MARCM clones expressing *PFK1-RNAi* or *PK-RNAi*. Under these conditions, the size of *FASN* ^*Δ24-23*^ cells, expressing either RNAi was hardly reduced in larvae fed a LCD (Fig. 5E,G,N). However, in larvae fed a 20%-SSD, the size of *FASN* ^*Δ24-23*^ cells remained unaffected when expressing *PFK1-RNAi* (Fig. 5F,N), but were dramatically reduced when expressing *PK-RNAi* (Fig. 5H,N). The phenotypic suppression produced by *PFK1-RNAi*, suggests that an intermediate metabolite, downstream of PFK1 (Fig. 4C), is responsible for the size reduction of *FASN* ^*Δ24-23*^ cells observed in SSD-fed larvae. Therefore, MARCM clones, expressing *glo1-RNAi* were analyzed. Interestingly, MARCM *FASN* ^*+*^ clones expressing only the *glo1-RNAi* were insensitive to sucrose supplementation (Fig. 5I-J,O). Nonetheless, *FASN* ^*Δ24-23*^ cells expressing *glo1-RNAi* exhibited an extreme size reduction in larvae fed LCD (Fig. 5K,O) accompanied by a severe decrease in nucleus size (see below). Furthermore, these clonal cells could not be observed when larvae were fed 20%-SSD, suggesting that these cells were eliminated during development. Conversely, *FASN* ^*Δ24-23*^ MARCM cells overexpressing *glo1* were of normal size in larvae fed a 20%-SSD (Fig. 5L,P). However, neither *FASN* ^*Δ24-23*^ MARCM cells in LCD-fed larvae, nor *FASN* ^*+*^ MARCM cells were affected in size by Glo1 overexpression (S6J–S6L,S6M Fig.). Together, these findings indicate that Glo1 can compensate for cell size reduction due to a sugar-dependent FA-synthesis defect, but is unlikely to promote cellular growth.

**Fig 6:**
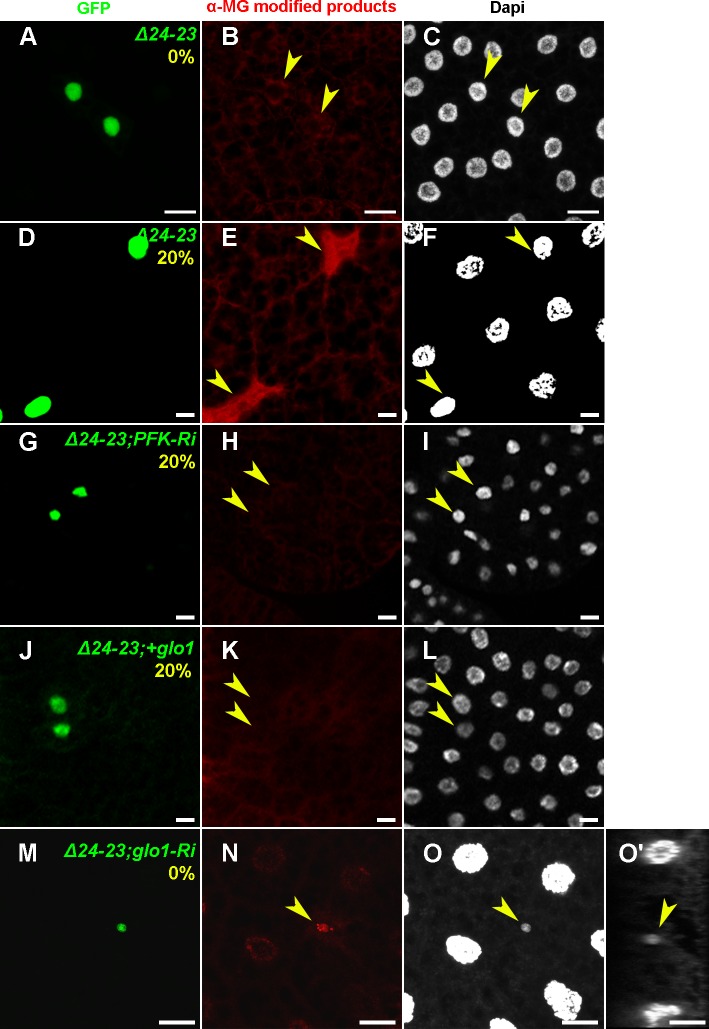
Cell-autonomous accumulation of MG-AGEs in *FASN* mutant FB cells. Immunodetection of MG-AGEs (B,E,H,K,N) and DAPI staining (C,F,I,L,O,O’) in FB from feeding L3 larvae containing MARCM clones labeled with GFP (A,D,G,J,M). (A-F) Accumulation of MG-AGEs in *FASN* ^*Δ24-23*^ MARCN clones of L3 larvae fed either a LCD (A-C) or a 20%-SSD (D-F). (G-L) Suppression of MG-AGE accumulation in *FASN* ^*Δ24-23*^ MARCN clones of L3 larvae fed a 20%-SSD by either *PFK1-RNAi* (G-I) or *UAS-glo1* (J-L). (M-O) Accumulation of MG-AGEs and extreme nuclear size reduction in *FASN* ^*Δ24-23*^ MARCN clones expressing *glo1-RNAi* of L3 larvae fed a LCD. (O’) is an orthogonal section of (O). Scale bars: 20μm. For each condition, at least 10 larvae were dissected.

Finally, an antibody to MG-derived AGEs (MG-AGEs) was used for immunostaining. In *FASN* ^*Δ24-23*^ clonal cells the amounts of MG-AGEs were barely detectable in larvae fed a LCD (Fig. 6A-C) but were dramatically increased in larvae fed a 20%-SSD (Fig. 6D-F). Importantly, increased MG-AGE levels induced by 20%-SSD were abolished in *FASN* ^*Δ24-23*^ MARCM clones expressing either *PFK1-RNAi* (Fig. 6G-I) or *UAS-glo1* (Fig. 6J-L). Furthermore, in larvae fed a LCD, *FASN* ^*Δ24-23*^ clones expressing *glo1-RNAi* exhibited a strong accumulation of MG-AGEs (Fig. 6M-O). Nucleus size in these clones, was also dramatically reduced (Fig. 6O,O’). Taken together, these findings indicate that FA synthesis and Glyoxalase activity cooperate in a cell-autonomous manner to neutralize the toxicity of dietary sugar, which may result in cellular growth defects or putative cell elimination.

## Discussion

**Fig 7:**
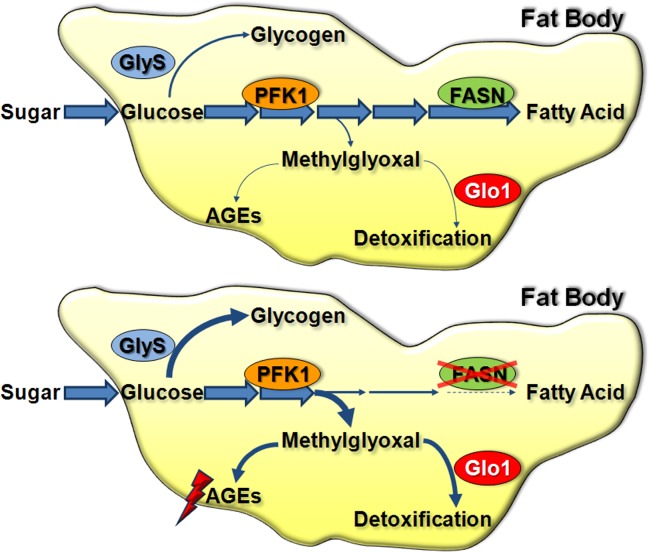
Glucose metabolic fate in the larval FB. (A) Under normal conditions, glucose that enters into FB cells is mainly used through glycolysis and FA synthesis for TAG storage. Glucose is also stored as glycogen. Small amounts of MG are formed; thus Glo1 activity is not critical. (B) In *FASN* mutant FB cells, FA synthesis is abolished and glycogen synthesis is increased. Excess of sugar provokes a dramatic increase in MG levels and elicits Glo1 activity.

In this study, we investigated the role of FA synthesis in regulating homeostasis in response to dietary sugar. To maintain tolerable levels of circulating sugars, organisms synthesize and store macromolecules in appropriate organs. In contrast to previous studies in insects, which report that the majority of TAGs stored in the FB are of dietary origin [29,30], we observed that in *Drosophila*, the larval FB is a lipogenic organ. However, in *FASN* ^*Δ24-23*^ mutant fed a beySD, TAG levels were decreased but not abolished. This indicates that as in mammalian hepatocytes and adipocytes [2,31,32], TAGs stored in the *Drosophila* larval FB originate from either food assimilation or *de novo* synthesis. Together, our findings confirm that metabolic pathways act within an integrative network to maintain homeostasis and support the notion that in term of post-feeding macromolecules storage (TAGs and glycogen), the *Drosophila* larval FB constitutes an alternative model for mammalian liver and adipose tissue (Fig. 7A).

Our *FASN* mutants are lethal at L1 stage, and this lethality can be recued by a beySD. Rescue of *FASN* ^*Δ24*^ but not of *FASN* ^*Δ24-23*^ mutants by soy lipid extracts likely reflects the strength of the mutation since *FASN* ^*CG3523*^ is still weakly expressed in the hypomorphic mutant. Consistently a SREBP mutant that down-regulates but does not abolished the expression of several FA anabolic enzymes including *FASN* ^*CG3523*^, could also be rescued by soy lipid extracts [33]. Rescue of the lethal phenotype by dietary lipids, as well as the minor phenotype observed in *FASN* ^*Δ24-23*^ clonal cells, suggests that neighboring cells or organs can provide FAs to those that are deficient. This may be achieved through lipophorin activity [34]. Intriguingly, we found that in contrast to other lipid-supplemented media, a mix of butter and egg yolk could rescue the *FASN* ^*Δ24-23*^ lethal phenotype. TAGs cannot be directly assimilated by enterocytes; first they require digestive lipases to cleave TAGs to di-acyl-glycerol (DAG), mono-acyl-2-glycerol (MAG) and free FAs (FFAs) [35,36]. In several mammalian species, lipids interact with bile acids to form micelles prior to enzyme cleavage and enterocyte absorption. However, it has been reported that in rats and human infants, FFAs may interact with calcium or magnesium ions to form soaps that are hardly assimilated [37,38,39]. In insects, lipid emulsifiers are poorly characterized although glycolipid or amino acid complexes are likely to be involved in lipid assimilation [40,41]. As egg yolk lipoproteins are highly efficient emulsifiers [42], they may help solubilize lipids, thereby favoring their absorption. The composition in FAs and their positions on the glycerol backbone vary depending on the origin of the TAGs. Regarding FA synthesis, *FASN* ^*Δ24-23*^ mutants are expected to lack palmitic acid. Analysis of various oils and fats, revealed that TAGs found in butter contain high quantities of palmitic acid in position *sn*-2 of the glycerol [36]. Thus, assimilation of MAGs resulting from butter digestion, are high in palmitic acid. Hence, it is possible that MAGs are better assimilated than FFAs in our *FASN* ^*Δ24-23*^ mutants. In order to fully understand the process of lipid absorption in *FASN* ^*Δ24-23*^ mutants, extensive analysis, including the precise measurement of ingested and excreted FAs, will be required.

Rescue of lethality of *FASN* mutant by a lipid-supplemented diet indicates that FA synthesis deficiency can be compensated for by an appropriate lipid diet. Previous studies in *Drosophila* have reported that the FA composition of the various lipid classes varies depending on the diet [43,44]. Here, we show that the relative FA composition of phospholipids is not significantly different in *FASN* ^*Δ24-23*^ rescued mutants and control animals fed a beySD. These findings not only confirm that diet contributes to phospholipid composition, but reveal that in the presence of an exogenous lipid supply, the essential FASN enzyme becomes dispensable for phospholipid synthesis. In contrast, sterol esters and TAGs exhibit variation in their FA composition. Compared to controls, TAGs from mutants contain less saturated FAs and more long chain unsaturated FA, suggesting that expression of desaturases and elongases [45] may be increased in *FASN* mutants. The high variability in TAG composition suggests that TAG structure is not a crucial parameter, which strengthens the notion that TAG synthesis constitutes a metabolic strategy to neutralize the potential toxicity of nutrients. Previous studies suggested that the fat tissue fulfills a protective role against excess sugar. In agreement with this, it has been shown that fat transplantation in lipoatrophic mice reverses T2D [46,47] and that in genetically induced obese mice, a decrease in adipose FASN expression is linked to T2D progression [48]. In addition, mice and flies with defects in ChREBP—a transcriptional activator of lipogenic enzyme expression—do not survive increases in dietary sugar levels [44,49,50]. However, it was unknown that FASN activity also protects against sugar toxicity. This finding is in contrast to a previous report which showed that in flies, lethality induced by ubiquitous expression of *FASN* ^*CG3523*^ *-RNAi* can be partially rescued by dietary sugar [50]. Here, we demonstrate that FA synthesis protects against dietary sugar at both a systemic and cell-autonomous level. The media used in our study contained low concentrations of digestible sugar, 64, 164 and 264 mg/ml for the LCD, 10%-SSD and 20%-SSD, respectively. In other studies, which used *Drosophila* larvae as a model for sugar tolerance, the concentration of digestible sugar was 86, 140 or 80 mg/ml for the low carbohydrate media and 377, 380 or 230 mg/ml for the sugar enriched media [27,50,51]. Importantly, while circulating sugar levels increase in *FASN* ^*Δ24-23*^ animals, these mutants do not exhibit a T2D-like phenotype and become insulin hypersensitive. Therefore, as previously suggested [44,49,50], disrupting FA synthesis provides a convenient model to investigate the effect of glucotoxicity independent of lipotoxicity.

Here, we provide evidence to propose that FASN and Glo1 cooperate both in a systemic and in a cell-autonomous manner to protect against the deleterious effect of dietary sucrose. Our findings indicate that when FA synthesis is very active, as in the FB of *Drosophila* larvae, Glo1 activity is dispensable in term of neutralizing the few toxic metabolites produced through sugar catabolism (Fig. 7A). Conversely, the detoxifying activity of Glo1 becomes critical when *FASN* activity is disrupted in the larval FB (Fig. 7B). Thus, the observed decrease in lipogenic enzyme expression in the adipose tissue of a diabetic mouse model [48], may require an increase in Glo1 activity. If lipogenic enzyme expression is also decreased in T2D patients, the increase in glycating agents [52] may result not only from an increase in circulating sugar but also from a decrease in FA synthesis. For a few decades, pathological damage induced by excess sugar was thought to be a consequence of AGE formation [53], a paradigm substantiated by recent studies on experimental diabetic nephropathy [9,10,54]. Consistent with a study in *Caenorhabditis elegans*, reporting that Glo1 overexpression protects against glucose toxicity [55], we show that manipulating Glo1 levels in the larval FB modulate a sugar-induced developmental delay. Studies in diabetic models and patients mostly focused on AGE levels in body fluids [56,57,58,59], although alterations to intracellular products have also been reported [60,61,62]. At the cellular level, *glo1* knockdown in FB cells induces a cell-autonomous phenotype, only when clones are also *FASN* deficient. This phenotype results in either an extreme reduction in cell size or elimination of cells, when larvae are fed LCD or SSD, respectively. The number of FB cells is determined during a proliferative phase at embryogenesis. During larval life, FB cells do not divide, but undergo a rapid cell growth phase [63,64]. The lack of a visible phenotype in *glo1*-deficient cells indicates that even when larvae are fed SSD, Glo1 does not affect the growth process of FB cells. In contrast, increasing quantities of sucrose in the food, even to moderate levels, induces a size reduction of *FASN* mutant cells. This phenotype is unlikely to depend directly on sugar since addition of moderate amounts of sucrose to food media does not markedly increase circulating sugar levels. In contrast, it is likely to directly depend on an increase of intracellular MG, since the cell size reduction is suppressed if *FASN* mutant cells are either deficient in *PFK1* or overexpressing *glo1* cDNA. In summary, our findings suggest that FASN activity is dispensable in sustaining cell growth but plays a key role in protecting against the potentially toxicity of MG produced through glycolysis.

In conclusion, we have demonstrated that FA synthesis constitutes a metabolic strategy to restrict the production of intermediate toxic molecules, suggesting that obesity is not a harmful process, as long as storage capacity is not overwhelmed. Furthermore, our study highlights the need for caution when using FA synthesis inhibitors to treat cancers and metabolic diseases, as they might provoke negative side effects.

## Materials and Methods

### Fly stocks and genetics

Fly strains: *P[tGPH]* [28], *daughterless(da)-gal4*, *Mef2-gal4*, *actin5C>CD2>gal4*,*UAS-GFP*, *P[w[+mC] = tubP-GAL80]LL10*,*P[ry[+t7*.*2] = neoFRT]40A*, *UAS-Dcr-2* (Bloomington Stock Center); Inducible RNA-interfering (*UAS-RNAi*) lines to *ACC* (VDRC 108631), *FASN* ^*CG3523*^ (VDRC 29349), *FASN* ^*CG3524*^ (VDRC 4290), *GlyS* (VDRC 35136), *glo1* (remobilized on chromosome III from VDRC 26832), *PFK1* (VDRC 3017), *PK* (remobilized on chromosome III from VDRC 49533); *FAS* ^*CG17374*^-*RNAi*, *svp-gal80*, *Cg-gal4* [21]. The P-element insertions (Exelixis collection) *P[XP]v(2)k05816* ^*d04154*^ and *P[XP]CG3523* ^*d06961*^ were used to generate deficiency as described [65]. All the fly lines were isogenized from single males in a *white* ^*1118*^ mutant (*w* ^*-*^) background. For clonal analysis *FASN* ^*Δ24-23*^ was over *SM5-TM6B*,*Tb* ^*-*^ balancers [21]; For survival and metabolic analyses, *FASN* mutants were balanced by a *CyO* GFP-labelled chromosome.

The results presented for ubiquitous or tissue-targeted *UAS-RNAi* lines—including the corresponding controls—were obtained with a *UAS-Dcr-2* that strengthens the RNAi effect. Developmental delays were evaluated from overnight egg collection and the number of prepupae formed was counted every morning. For each assay, several tubes were collected, overcrowded tubes were discarded and the numbers of prepupae were pooled. As some of the transgenes used in the genetic combinations were homozygous lethal, all the lines (driver, RNAi, *w* ^*-*^ control) were balanced with co-segregating *SM5-TM6B*,*Tb* ^*-*^ balancers that lead to non-mendelian offspring distribution. Therefore, for each assay, the number of RNAi-expressing *Tb* ^*+*^ larvae was divided by the final number of *Tb* ^*-*^ larvae and all assays were normalized to the control ratio. For controls, a similar calculation was done from the offspring of driver females crossed with *w* ^*-*^;*SM5-TM6B* males and this control ratio was adjusted to reach 100%.

### Molecular biology

To generate the overexpressing lines, the locus of *FAS* ^*CG3523*^, and *FAS* ^*CG3524*^ were recovered by gap repair and the endogenous promoter replaced by a UAST [66]. *glo1* cDNA was amplified from GH24818 (DGRC) and cloned into the pUAST vector. Plasmid constructs were injected by BestGene.

RT-Q-PCR were performed as previously described [21] using the following primers:

*FASN* ^*CG3523*^ (5’-F CTTCTTCATTTCCCCGA-3’ and 5’-CGAAGGAGTATCCGGC-3’)

*FASN* ^*CG3524*^ (5’-CTTTGACAATATGCTCTAC-3’ and 5’-AAGTCCGGAGTGTCCAG-3’)

*FASN* ^*CG17374*^ (5’-F ATCAGCTCCAACCTCTAC-3’ and 5’-GGGCTACATGCAAGTCT-3’)

*ACC* (5’-TTGGGAAACTCATTCGTG-3’ and 5’-CCAGGACCTTGGCATTA-3’)

*GlyS* (5’-CCCCTCATACTACGAGC-3’ and 5’-CGATATAGCGGCGATCC-3’)

### Immunocytochemistry

Flip-out clones were performed as described [21]. MARCM clones [67] were heat-shock induced at 4–6h embryogenesis and the larvae were allowed to grow on various sucrose-supplemented media until mid/late L3 stage. FB were dissected as described [21] but fixed with 3.7% formaldehyde in PBT (PBS 0.1% Tween20). FB were stained with Phalloidin-Rhodamine B (sigma) at 625 ηg/ml for 2h at RT, extensively washed and mounted in DABCO (sigma). Relative cell size was expressed as a ratio, clonal:neighboring control cells, which was estimated using the image-j software. The insulin responsive assay was performed as described [27]. tGPH quantification was measured in squares (10X10 pixels) positioned either at the membrane or at the nuclei. Measurements for each assay were recorded from 8 cells taken from 2 different FBs. For each cell, the maximum fluorescence intensity at the membrane was divided by the maximum fluorescence intensity at the nucleus and the mean ratio was plotted (S4B Fig.). Nile Red staining was performed as previously described [21]. For MG-AGE Immunostaining, dissected FB were fixed as described above, but blocked for 20 mn in PBS containing 0.1% Triton X100 and 2% bovine serum albumin. Samples were incubated overnight at 4°C with diluted (1:400) MG-AGE antibody (Cell Biolabs), extensively washed and incubated for 2h at room temperature with secondary antibody and DAPI in the blocking solution. Samples were finally washed in PBT and mounted in DABCO. Image acquisitions were obtained using a Nikon TE2000-U or a Leica SP8 confocal laser-scanning microscopes.

### Metabolic measurements

TAGs, protein, glucose, trehalose and glycogen measurements were performed as previously described [21]. To measure circulating sugar, 6μl samples of hemolymph were collected from 20 to 30 bled L3 larvae. For AGE measurement, 5 samples of 10 L3 larvae were washed in PBS and crushed at 4°C in a Precellys 24; extracts were cleared 10 mn at 4° C in a microfuge at maximum speed. Extracts were diluted 100X in PBS and 100 μL of this diluted extract were treated with an ELISA kit (Cell Biolabs STA-317). AGEs estimation evaluated from spectrophotometric dosage at 450nm, was normalized to the protein concentration of each sample. To measure feeding rates, food media was tinted with 0,1% brilliant blue FCF. 3 samples of 10 L3 larvae were collected, frozen, extracted in 200μl water and centrifuged for 7min at maximum speed. The final volume was adjusted to 800μl and measured at 629ηm. Lipidomics were performed in triplicates of 100 mg 0–5h prepupae. Lipids were extracted and analyzed by GC-MS as described [68].

### Statistical analysis

Statistical analyses were performed with R version 3.0.2 [69]. Error bars in figures stand for empirical standard deviations measured independently from the replicates in each category. Significance for the statistical tests was coded in the following way based on the p-values: ***: 0 < p < 0.001; **: 0.001 < p < 0.01; *: 0.01 < p < 0.05. In all the graphs, the error bars represent the standard deviations.

For S3 Table (corresponding to Fig. 1C, 1D, 4D, 4E, and S5A–S5C Fig.), the effect of the genotype was tested with one-way ANOVAs on developmental rates. Developmental rates (in units of days-1) were computed as the inverse of developmental duration to pupation. Lethality (evaluated for each developmental curve and corrected with the lethality rates for control measured in S5D Fig.) was accounted for by including a corresponding number of (unobserved) lethal events (individuals with a developmental rate of 0). Since all nine ANOVAs detected a significant effect of the genotype, pairwise comparisons between genotypes were tested with a post-hoc Tukey "Honest significant difference" test [70] for each sub-figure, and the biologically-relevant comparisons are reported.

## Supporting Information

S1 Fig Expression and function of the FASN genes in larvae. (A) Quantitative RT-Q-PCR (means calculated from 3 samples of 10 feeding L3 larvae) to determine *FASN* ^*CG3523*^, *FASN* ^*CG3524*^ and *FASN* ^*CG17374*^ levels was performed on either the internal organs (black bars) or the left over carcass (grey bars) separated of *w* ^*-*^ L3 larvae. (B-D) The tracheal trunks are filled of air in *w* ^*-*^ control larvae (B), whereas ubiquitous expression of *FASN* ^*CG17374*^ *-RNAi* induces tracheal flooding (arrowhead in C), a phenotype which is rescued when the *svp-gal80* transgene is expressed (D). Tracheal phenotypes have been analyzed in the progeny of at least 3 distinct crosses.
(TIF)

S2 Fig Metabolic modification induced by GlyS, ACC and *FASN* ^CG3523^ knock down in FB and muscles. (A) Total TAG levels in larvae expressing RNAi to *FASN* ^*CG3523*^, *FASN* ^*CG3524*^ or *FASN* ^*CG17374*^ within their FB. (B-G) RNAi to *GlyS*, *ACC* and *FASN* ^*CG3523*^ were induced alone or in combination with the *Cg-gal4* driver (FB), the *Mef2-gal4* driver (Muscles) or both drivers together. (B) Mean weight (mg) of 0–5h prepupal female (n = 20). An ANOVA shows an effect of genotype (S1 Table) and a significant interaction between genotype and targeted tissue (S1A Table). The strongest interactions are observed in the combination of double-RNAi with dual-organ knockdowns (-0.37mg; T-test: p-value < 10^-13^). (C-G) Total concentration of TAGs (C), glycogen (D), trehalose (E), glucose (G) and proteins (H) in 0–5h prepupae. (C) TAG levels dramatically decrease for *ACC* and *FASN* ^*CG3523*^ knockdowns in the FB. (D) Glycogen levels increase for single knockdown of either *ACC* or *FASN* ^*CG3523*^ in the FB. Glycogen levels significantly decrease for *GlyS* knockdown in either the FB (p-value = 10^-5^) or the muscles (p-value = 4.10^-4^). When the *GlyS-RNAi* is expressed in both tissues, the decrease in glycogen levels is compatible with an additive effect of FB and muscles (no significant interaction in a linear model: p-value = 0.11). (E) The decreases in trehalose levels decrease follow the decreases in glycogen levels. (F) The correlation between glycogen and thehalose levels is significantly positive (r = 0.70, p-value < 10^-11^). (G-H) Glucose (G) and protein (H) levels do not exhibit dramatic perturbations. The values represent the concentration of each metabolite in μg per mg of prepupae. Controls (Co) correspond to offspring resulting from a cross between driver females and *w* ^*-*^ control males. All tested animals expressed *UAS-Dcr2*. TAGs and protein values are means calculated from 5 samples of 150 mg prepupae; glucose, trehalose and glycogen values are means calculated from 4 samples of 500 mg prepupae. Experiments repeated twice. Color code for (B-H) is indicated at the bottom of the figure. The panels display the significant stars for the only comparisons mentioned in the result section.
(TIF)

S3 Fig Lipid profiles of phospholipid in *FASN* null mutants. FA profiles of the lipid classes from either *w* ^*-*^ control (Co) or *FASN* ^*Δ24-23*^ (*Δ24-23*) mutant animals fed a beySD. Relative abundance of saturated and unsaturated FAs in total phospholipid (A), phophatidylethanolamine (B), phophatidylcholine (C), phophatidylinositol (D), phophatidylserine (E) and sphingolipid (F). Profiles represent means calculated from 3 samples of 100 mg 0–5h prepupae.
(TIF)

S4 Fig Characterization of *FASN* mutants. (A) Survival until metamorphosis or early larval lethality (**†**) of *FASN* ^*Δ24*^ hypomorph mutants in the presence (10%) or absence (0%) of sucrose supplementation; *w* ^*-*^ control (Co) and *FASN* ^*Δ24*^ (*Δ24*) animals were fed a LCD (Lipids 0%) or the same media complemented with soy lipid extracts (Lipids 4%). For each condition, groups of 80 newly hatched larvae were placed in 3 tubes; values represent the means of larvae surviving to metamorphosis. (B) Quantification of tGPH intensity in FB explants from *w* ^*-*^ control (Co) and *FASN* ^*Δ24-23*^ (*Δ24-23*) animals fed either a LCD (0%) or a 10%-SSD (10%). FB explants were incubated (+) or not (-) with insulin.
(TIF)

S5 Fig Sugar metabolism defects. (A-C) Developmental delay evaluated at puparium formation of larvae fed a LCD (A), a 10%-SSD (B) or a 20%-SSD (C). The *Cg-gal4* driver was used to express RNAi to *PFK1* or *PK* within the FB. Controls (Co) are the progeny resulting from a cross between *Cg-gal4* females and *w* ^*-*^ balanced males. In this experiment (A-C), each curve represents at least 300 animals. (D) Lethality of *w* ^*-*^ control larvae fed a LCD (0%), a 10%-SSD (10%) or a 20%-SSD (20%). For each condition, groups of 80 newly hatched larvae were placed in 5 tubes. values represent the means of larvae surviving to metamorphosis. Note that basal lethality happens for animal fed a LCD or a 10%-SSD that may result from larval handling at hatching phase. When fed a 20%-SSD *w* ^*-*^ control larvae exhibit an 18% increase in larval lethality.
(TIF)

S6 Fig Clonal analysis in FB cells. (A-F) Phalloidin staining of FB dissected from feeding L3 larvae containing flip-out clones labeled with GFP. At the top right corner of each image, the genotype of the clonal cells and the % sucrose supplementation are shown in green and yellow, respectively. *FASN* ^*CG3523*^ *-RNAi* (A-B) and *PK-RNAi* (C-D) flip-out cells are of roughly normal size when animals are fed a LCD (A-C), but are reduced in size when fed a 20%-SSD (B,D). (E-F) *PFK1-RNAi* flip-out cells are of roughly normal size when animals are fed either a LCD (E) or a 20%-SSD (F). (G-I) Nile red staining of *FASN* ^*Δ24-23*^ mutant cells dissected from larvae fed a LCD (G), a 10%-SSD (H) or a 20%-SSD (I). (J-L) Phalloidin staining of *FASN* ^*Δ24-23*^ (J) or *FASN* ^*+*^ (K-L) MARCM clones expressing *UAS-glo1* in FB dissected from L3 larvae that were fed either a LCD (J-K) or a 20%-SSD (L). Scale bars: 20μm. (M) Size ratio between at least ten clonal cells and the neighbouring control cells, as shown in (A-F,J-L). For each condition, at least 10 larvae were dissected.
(TIF)

S1 Table Statistical analysis related to S2B Fig. The effects of the targeted tissue (Organ) and of the downregulated genes (Gen) were tested by two distinct analyses of variance. (**A**) Two-way ANOVA considering six genotypes (as in S2B Fig.), three targeted organs, as well as the genotype x organ interaction. (**B-D**) The data set was split according to the targeted organ—FB(**B**), muscle(**C**) or both(**D**)—, and a post-hoc Tukey test was run within each organ to test for pairwise differences across genotypes; tables report adjusted p-values. (**E**) Four-way ANOVA in which each downregulated gene was treated independently, including pairwise interactions between downregulated genes to test for epistasis. Df: degree of freedom; Sum Sq: sum of squares; Mean Sq: mean squares.
(DOC)

S2 Table Feeding media recipes. Quantities of each product (g) per 100ml of feeding media are indicated in the top part of the table. The amounts of potential digestible sugar are indicated for each product in parenthesis. (nd) none digestible sugar. Total amounts of digestible sugar are indicated in bold. For SSDs, 5g, 10g or 20g of sucrose were added to 100ml of LCD. The media used in the various experiments are indicated in the bottom part of the table.
(DOC)

S3 Table Statistical analysis. Statistical analysis was performed in R version 3.0.2, by running linear models with genotype coded as a fixed factor. “Diff DR” stands for the estimated difference in developmental rates between genotypes. Developmental rates (in units of days^-1^) were computed as the inverse of developmental duration to pupation. Lethality (evaluated for each developmental curve and corrected with the lethality rates for *w* ^*-*^ control measured in S5D Fig.) was accounted for by including a corresponding number of (unobserved) lethal events (individuals with a developmental rate of 0).
(DOC)

S4 Table Partial genetic rescue of *FASN* mutants. Column 1 lists the genetic background for *FASN* that is either wild type (*w* ^*-*^) hypomorph (*FASN* ^*Δ24*^) or null (*FASN* ^*Δ24-23*^). Column 2 and 3 indicate the usage of UAS trangenes and driver, respectively. Column 4 reports the stage of lethality (**†**) or the oldest developmental stage reached. L1-L3: larval stages; pp: pupal stage; ad: adult stage. Column 5 and 6 indicates the percentage of rescued animals reaching pupal and adult stages, respectively. Each test has been repeated at least 3 times. For quantification of genetic rescue, *FASN* mutants were combined with UAS transgenes in front of co-segregating *SM5-TM6B*,*Tb* ^*-*^ balancers. For *UAS-FASN* ^*CG3524*^ rescue experiments, balanced flies were crossed together and groups of 50 *Tb* ^*+*^ L2 larvae were placed in 3 tubes, in order to determine the percentage reaching pupal rescue. For *UAS-FASN* ^*CG3523*^ rescue experiments, balanced females were mated with homozygous males. The progeny of this cross follows a mendelian distribution, with 50% of *Tb* ^*+*^ and 50% of *Tb* ^*-*^ animals. Therefore, the percentage of rescue correspond to the number of *Tb* ^*+*^ rescued animals relative to the number of *SM5-TM6B*,*Tb* ^*-*^ balanced animals.
(DOC)

S5 Table Partial feeding rescue of *FASN* mutants. Quantities (g) of each lipid nutrient per 100ml of feeding media are indicated in the 5 top lines of the table. The various lethal stages reached by *FASN* ^*Δ24*^ (***Δ24***) and *FASN* ^*Δ24-23*^ (***Δ24-23***) mutant animals are indicated in the 2 bottom lines. L1-L3: larval stages; pp: pupal stage; ad: adult stage. Note that the stage of survival is variable for all rescuing media. Each test has been repeated at least 3 times. For the lipid-feeding rescue of *FASN* ^*Δ24-23*^ mutants, groups of 30 homozygous L1 larvae were placed in 5 tubes to determine the percentage of larval, pupal and adult survival.
(DOC)
