# Supplementary figures and images for: Fatty Acid Synthase Cooperates with Glyoxalase 1 to Protect against Sugar Toxicity
Source: PLoS Genet. 2015 Feb 18;11(2):e1004995. doi: 10.1371/journal.pgen.1004995 (PMC4334898; doi:10.1371/journal.pgen.1004995)

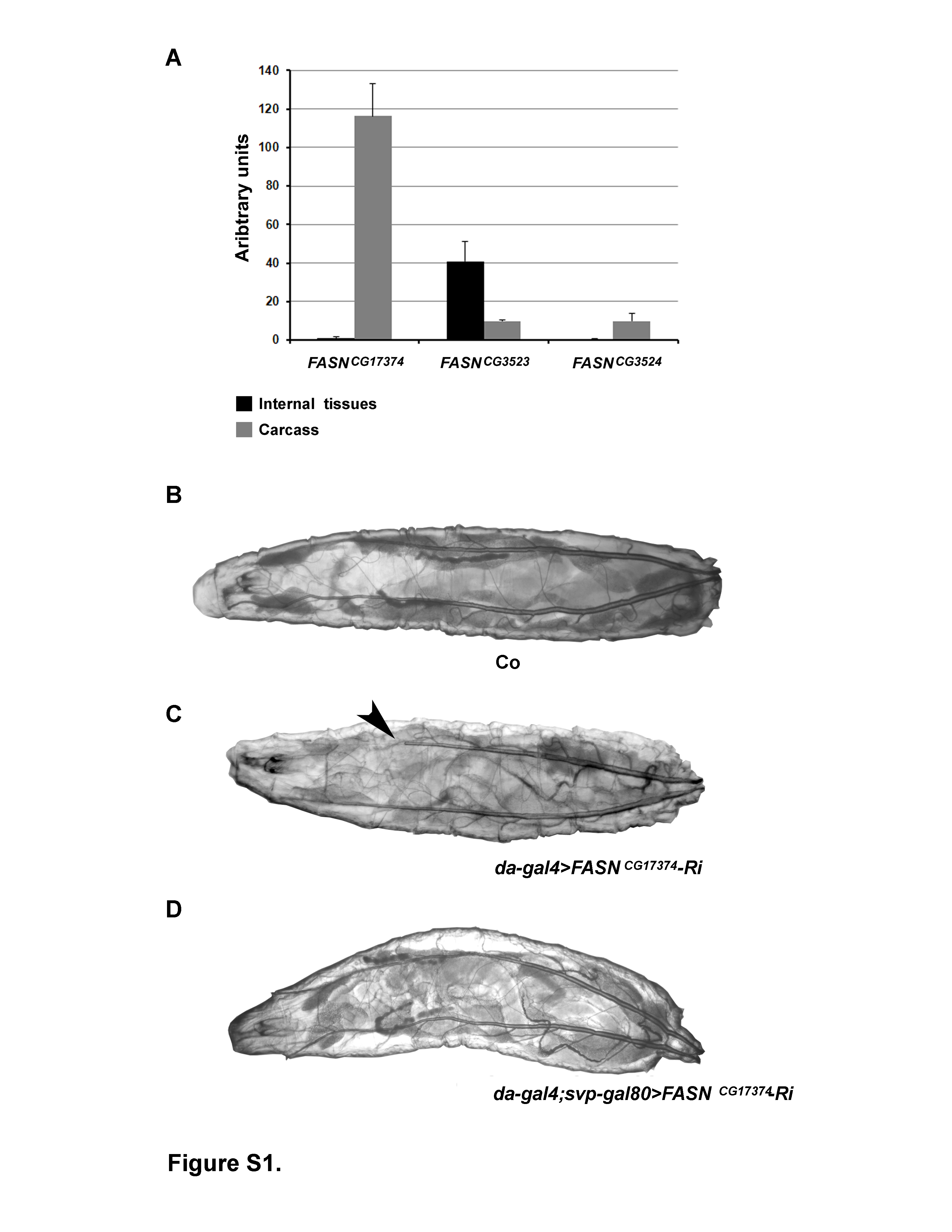

Supplement: S1 Fig — (A) Quantitative RT-Q-PCR (means calculated from 3 samples of 10 feeding L3 larvae) to determine FASN CG3523, FASN CG3524 and FASN CG17374 levels was performed on either the internal organs (black bars) or the left over carcass (grey bars) separated of w - L3 larvae. (B-D) The tracheal trunks are filled of air in w - control larvae (B), whereas ubiquitous expression of FASN CG17374 -RNAi induces tracheal flooding (arrowhead in C), a phenotype which is rescued when the svp-gal80 transgene is expressed (D). Tracheal phenotypes have been analyzed in the progeny of at least 3 distinct crosses. (TIF) [file pgen.1004995.s001.tif]

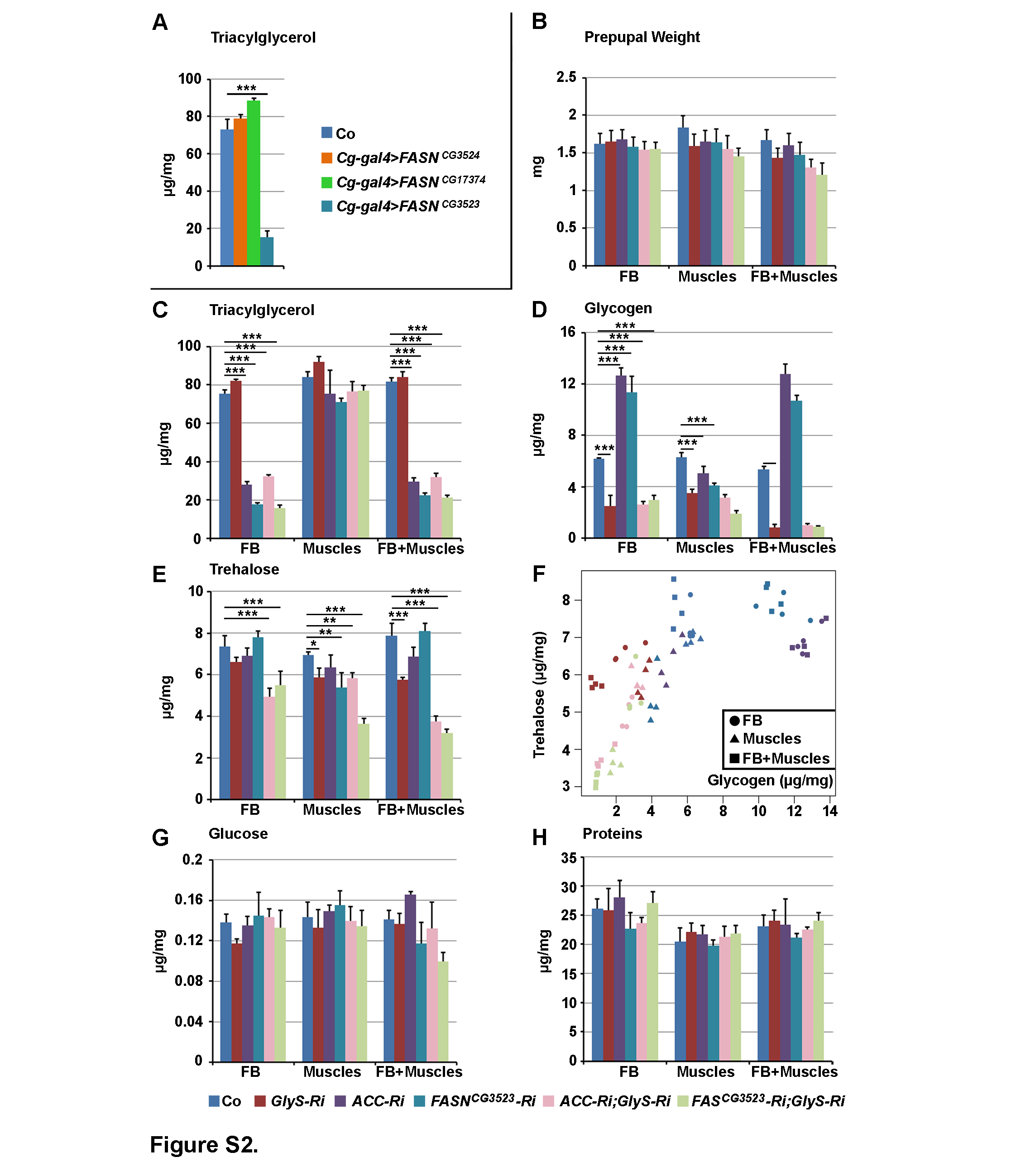

Supplement: S2 Fig — (A) Total TAG levels in larvae expressing RNAi to FASN CG3523, FASN CG3524 or FASN CG17374 within their FB. (B-G) RNAi to GlyS, ACC and FASN CG3523 were induced alone or in combination with the Cg-gal4 driver (FB), the Mef2-gal4 driver (Muscles) or both drivers together. (B) Mean weight (mg) of 0–5h prepupal female (n = 20). An ANOVA shows an effect of genotype (S1 Table) and a significant interaction between genotype and targeted tissue (S1A Table). The strongest interactions are observed in the combination of double-RNAi with dual-organ knockdowns (-0.37mg; T-test: p-value < 10-13). (C-G) Total concentration of TAGs (C), glycogen (D), trehalose (E), glucose (G) and proteins (H) in 0–5h prepupae. (C) TAG levels dramatically decrease for ACC and FASN CG3523 knockdowns in the FB. (D) Glycogen levels increase for single knockdown of either ACC or FASN CG3523 in the FB. Glycogen levels significantly decrease for GlyS knockdown in either the FB (p-value = 10-5) or the muscles (p-value = 4.10-4). When the GlyS-RNAi is expressed in both tissues, the decrease in glycogen levels is compatible with an additive effect of FB and muscles (no significant interaction in a linear model: p-value = 0.11). (E) The decreases in trehalose levels decrease follow the decreases in glycogen levels. (F) The correlation between glycogen and thehalose levels is significantly positive (r = 0.70, p-value < 10-11). (G-H) Glucose (G) and protein (H) levels do not exhibit dramatic perturbations. The values represent the concentration of each metabolite in μg per mg of prepupae. Controls (Co) correspond to offspring resulting from a cross between driver females and w - control males. All tested animals expressed UAS-Dcr2. TAGs and protein values are means calculated from 5 samples of 150 mg prepupae; glucose, trehalose and glycogen values are means calculated from 4 samples of 500 mg prepupae. Experiments repeated twice. Color code for (B-H) is indicated at the bottom of the figure. The panels disp [file pgen.1004995.s002.tif]

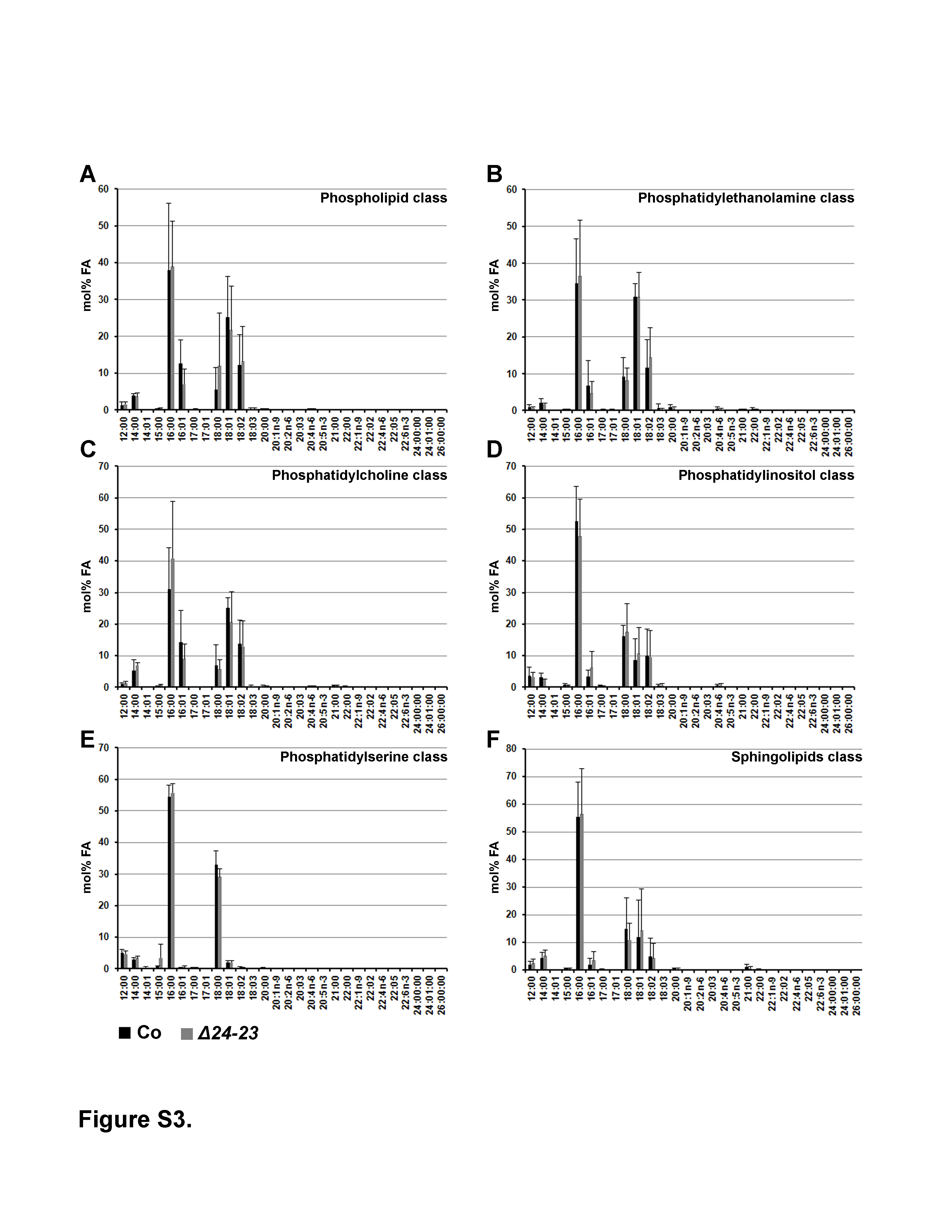

Supplement: S3 Fig — FA profiles of the lipid classes from either w - control (Co) or FASN Δ24-23 (Δ24-23) mutant animals fed a beySD. Relative abundance of saturated and unsaturated FAs in total phospholipid (A), phophatidylethanolamine (B), phophatidylcholine (C), phophatidylinositol (D), phophatidylserine (E) and sphingolipid (F). Profiles represent means calculated from 3 samples of 100 mg 0–5h prepupae. (TIF) [file pgen.1004995.s003.tif]

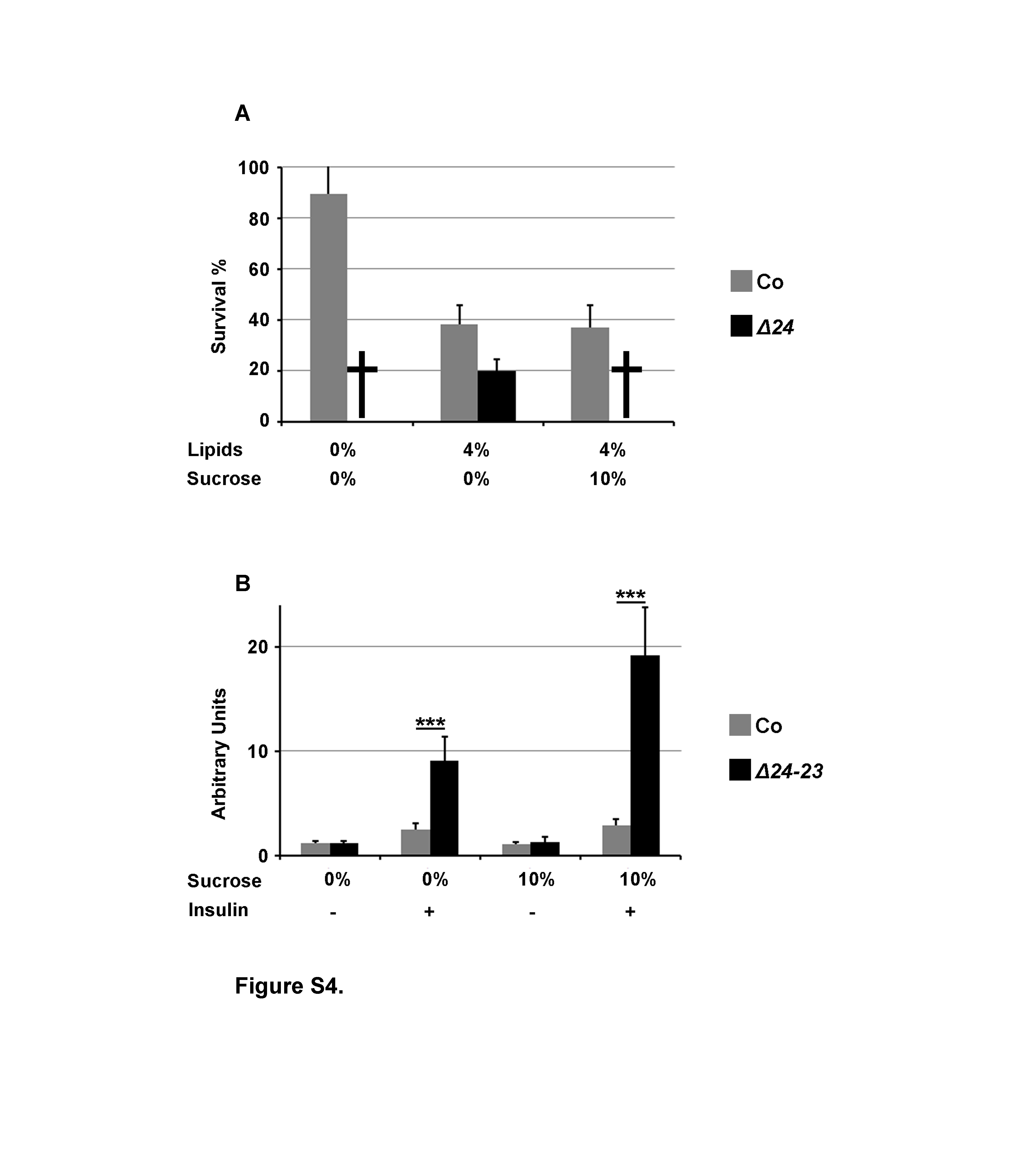

Supplement: S4 Fig — (A) Survival until metamorphosis or early larval lethality (†) of FASN Δ24 hypomorph mutants in the presence (10%) or absence (0%) of sucrose supplementation; w - control (Co) and FASN Δ24 (Δ24) animals were fed a LCD (Lipids 0%) or the same media complemented with soy lipid extracts (Lipids 4%). For each condition, groups of 80 newly hatched larvae were placed in 3 tubes; values represent the means of larvae surviving to metamorphosis. (B) Quantification of tGPH intensity in FB explants from w - control (Co) and FASN Δ24-23 (Δ24-23) animals fed either a LCD (0%) or a 10%-SSD (10%). FB explants were incubated (+) or not (-) with insulin. (TIF) [file pgen.1004995.s004.tif]

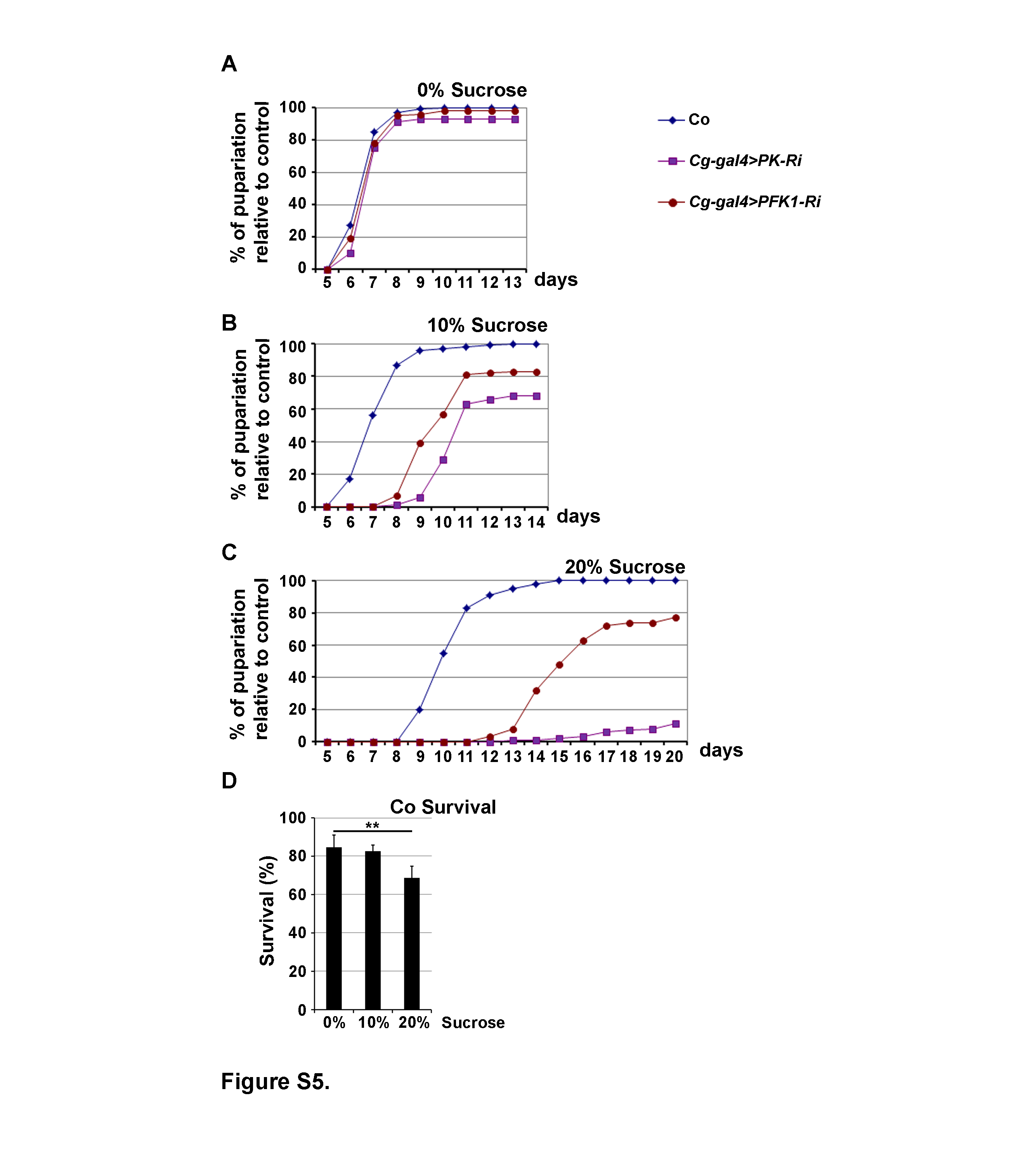

Supplement: S5 Fig — (A-C) Developmental delay evaluated at puparium formation of larvae fed a LCD (A), a 10%-SSD (B) or a 20%-SSD (C). The Cg-gal4 driver was used to express RNAi to PFK1 or PK within the FB. Controls (Co) are the progeny resulting from a cross between Cg-gal4 females and w - balanced males. In this experiment (A-C), each curve represents at least 300 animals. (D) Lethality of w - control larvae fed a LCD (0%), a 10%-SSD (10%) or a 20%-SSD (20%). For each condition, groups of 80 newly hatched larvae were placed in 5 tubes. values represent the means of larvae surviving to metamorphosis. Note that basal lethality happens for animal fed a LCD or a 10%-SSD that may result from larval handling at hatching phase. When fed a 20%-SSD w - control larvae exhibit an 18% increase in larval lethality. (TIF) [file pgen.1004995.s005.tif]

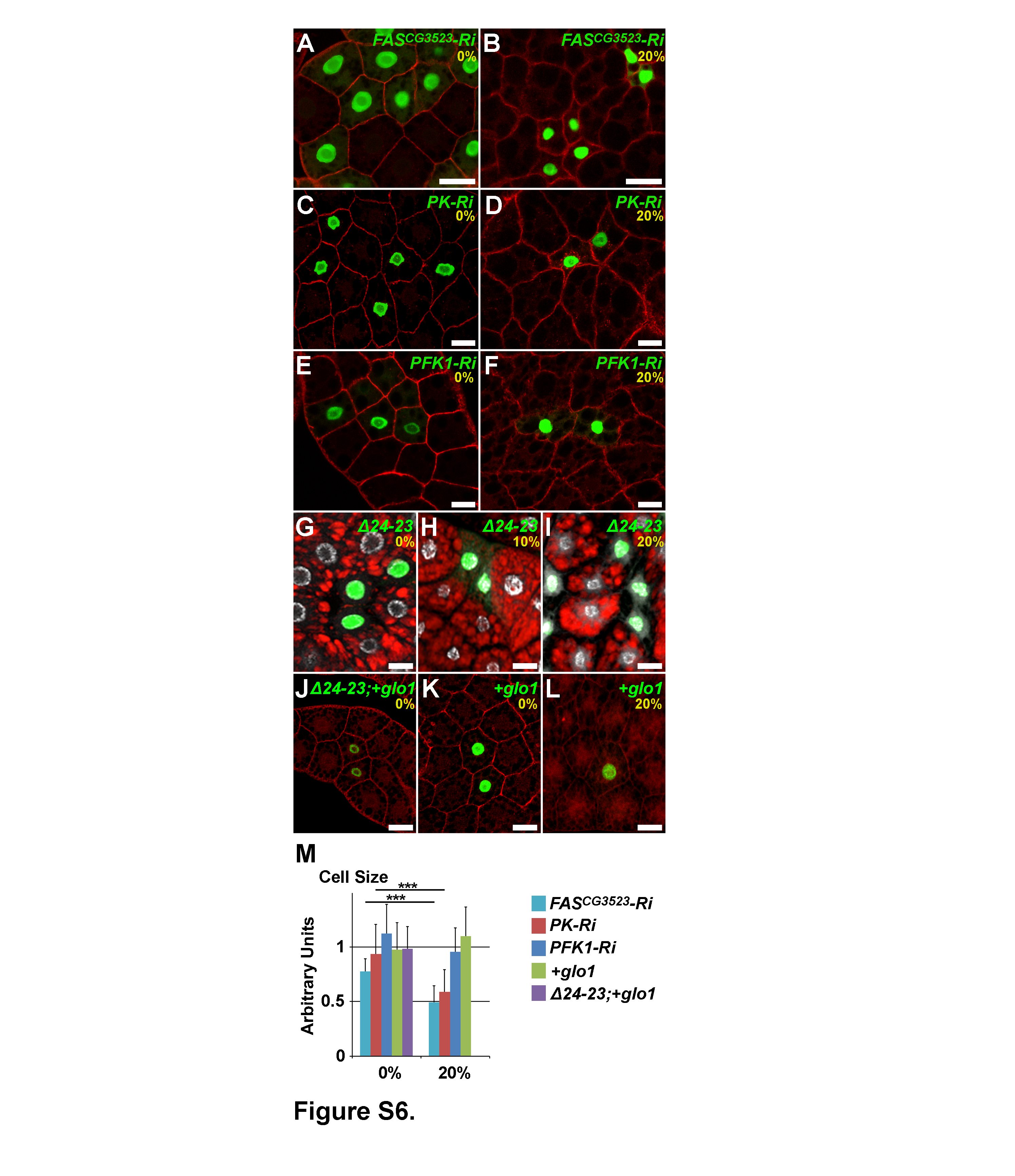

Supplement: S6 Fig — (A-F) Phalloidin staining of FB dissected from feeding L3 larvae containing flip-out clones labeled with GFP. At the top right corner of each image, the genotype of the clonal cells and the % sucrose supplementation are shown in green and yellow, respectively. FASN CG3523 -RNAi (A-B) and PK-RNAi (C-D) flip-out cells are of roughly normal size when animals are fed a LCD (A-C), but are reduced in size when fed a 20%-SSD (B,D). (E-F) PFK1-RNAi flip-out cells are of roughly normal size when animals are fed either a LCD (E) or a 20%-SSD (F). (G-I) Nile red staining of FASN Δ24-23 mutant cells dissected from larvae fed a LCD (G), a 10%-SSD (H) or a 20%-SSD (I). (J-L) Phalloidin staining of FASN Δ24-23 (J) or FASN + (K-L) MARCM clones expressing UAS-glo1 in FB dissected from L3 larvae that were fed either a LCD (J-K) or a 20%-SSD (L). Scale bars: 20μm. (M) Size ratio between at least ten clonal cells and the neighbouring control cells, as shown in (A-F,J-L). For each condition, at least 10 larvae were dissected. (TIF) [file pgen.1004995.s006.tif]
