# Supplementary material for: Fatty Acid Synthase Cooperates with Glyoxalase 1 to Protect against Sugar Toxicity
Source: PLoS Genet. 2015 Feb 18;11(2):e1004995. doi: 10.1371/journal.pgen.1004995 (PMC4334898; doi:10.1371/journal.pgen.1004995)
Supplement: S1 Table — The effects of the targeted tissue (Organ) and of the downregulated genes (Gen) were tested by two distinct analyses of variance. (A) Two-way ANOVA considering six genotypes (as in S2B Fig.), three targeted organs, as well as the genotype x organ interaction. (B-D) The data set was split according to the targeted organ—FB(B), muscle(C) or both(D)—, and a post-hoc Tukey test was run within each organ to test for pairwise differences across genotypes; tables report adjusted p-values. (E) Four-way ANOVA in which each downregulated gene was treated independently, including pairwise interactions between downregulated genes to test for epistasis. Df: degree of freedom; Sum Sq: sum of squares; Mean Sq: mean squares. (DOC) [file pgen.1004995.s007.doc]

| **A** | **Df** | **Sum Sq** | **Mean Sq** | **F value** | **p-value** |
| --- | --- | --- | --- | --- | --- |
| Organ | 2 | 247.3 | 123.64 | 60.616 | 0.000*** |
| Gen | 5 | 372.6 | 74.52 | 36.537 | 0.000*** |
| Organ x Gen | 10 | 189.1 | 18.91 | 9.273 | 0.000*** |
| Residuals | 429 | 875.0 | 2.04 |  |  |

| **B** | *Co* | *GlyS* | *ACC* | *FASN^3523^* | *GlyS; ACC* |
| --- | --- | --- | --- | --- | --- |
| *GlyS* | 0.895 |  |  |  |  |
| *ACC* | 0.480 | 0.941 |  |  |  |
| *FASN^3523^* | 0.856 | 0.273 | 0.092 |  |  |
| *GlyS; ACC* | 0.775 | 0.186 | 0.06 | 1 |  |
| *GlyS; FASN^3523^* | 0.145 | 0.007 | 0.003 | 0.888 | 0.914 |

| **C** | *Co* | *GlyS* | *ACC* | *FASN^3523^* | *GlyS; ACC* |
| --- | --- | --- | --- | --- | --- |
| *GlyS* | 0.000 |  |  |  |  |
| *ACC* | 0.008 | 0.853 |  |  |  |
| *FASN^3523^* | 0.003 | 0.956 | 0.999 |  |  |
| *GlyS; ACC* | 0.000 | 0.098 | 0.001 | 0.005 |  |
| *GlyS; FASN^3523^* | 0.000 | 0.960 | 0.254 | 0.459 | 0.360 |

| **D** | *Co* | *GlyS* | *ACC* | *FASN^3523^* | *GlyS; ACC* |
| --- | --- | --- | --- | --- | --- |
| *GlyS* | 0.000 |  |  |  |  |
| *ACC* | 0.451 | 0.000 |  |  |  |
| *FASN^3523^* | 0.000 | 0.994 | 0.000 |  |  |
| *GlyS; ACC* | 0.000 | 0.000 | 0.000 | 0.000 |  |
| *GlyS; FASN^3523^* | 0.000 | 0.071 | 0.000 | 0.207 | 0.211 |

| **E** | **Df** | **Sum Sq** | **Mean Sq** | **F value** | **p-value** |
| --- | --- | --- | --- | --- | --- |
| Organ | 2 | 247.3 | 123.64 | 51.004 | 0.000*** |
| Gen.*GlyS* | 1 | 210.3 | 210.34 | 86.771 | 0.000*** |
| Gen.*ACC* | 1 | 2.2 | 2.16 | 0.890 | 0.346 |
| Gen.*FASN^3523^* | 1 | 146.5 | 146.53 | 60.447 | 0.000*** |
| Gen.*GlyS:* Gen. *ACC* | 1 | 13.3 | 13.30 | 5.487 | 0.020* |
| Gen.*GlyS:* Gen. *FASN^3523^* | 1 | 0.3 | 0.29 | 0.120 | 0.730 |
| Residuals | 439 | 1064.2 | 2.42 |  |  |

**Table S1.**
