# Supplementary material for: Fatty Acid Synthase Cooperates with Glyoxalase 1 to Protect against Sugar Toxicity
Source: PLoS Genet. 2015 Feb 18;11(2):e1004995. doi: 10.1371/journal.pgen.1004995 (PMC4334898; doi:10.1371/journal.pgen.1004995)
Supplement: S2 Table — Quantities of each product (g) per 100ml of feeding media are indicated in the top part of the table. The amounts of potential digestible sugar are indicated for each product in parenthesis. (nd) none digestible sugar. Total amounts of digestible sugar are indicated in bold. For SSDs, 5g, 10g or 20g of sucrose were added to 100ml of LCD. The media used in the various experiments are indicated in the bottom part of the table. (DOC) [file pgen.1004995.s008.doc]

| **Media** | **Standard** | **LCD** | **Soy lipid** | **Mixed lipid** |
| --- | --- | --- | --- | --- |
| *Agar* | 1g (nd) | 1g (nd) | 1g (nd) | 1g (nd) |
| *Polenta* |  | 6g (4.8) | 6g (4.8) | 6g (4.8) |
| *Corn meal* | 8g (5.9) |  |  |  |
| *Yeast* | 8g (3.2) | 4g (1.6) | 4g (1.6) | 4g (1.6) |
| *Sucrose* |  |  |  |  |
| *Digestible sugar* | **9.1g** | **6.4g** | **6.4g** | **6.4g** |
| *Soy lipid extract* |  |  | 4g |  |
| *Butter* |  |  |  | 2.2g |
| *Egg yolk* |  |  |  | 2.5g |
|  |  |  |  |  |
| **Utilization** |  |  |  |  |
| Table 1 | **X** |  |  |  |
| Figure 1 |  | **X** |  |  |
| Figure 2 | **X** | **X** |  | **X** |
| Figure 3 |  |  |  | **X** |
| Figure 4 |  | **X** |  | **X** |
| Figure 5 |  | **X** |  |  |
| Figure 6 |  | **X** |  |  |
| Table S4 | **X** |  |  |  |
| Table S5 |  | **X** | **X** | **X** |
| Figure S1 | **X** |  |  |  |
| Figure S2 | **X** |  |  |  |
| Figure S3 |  |  |  | **X** |
| Figure S4 |  |  | **X** |  |
| Figure S5 | **X** | **X** |  |  |
| Figure S6 |  | **X** |  |  |

**Table S2.**
