# Supplementary material for: Fatty Acid Synthase Cooperates with Glyoxalase 1 to Protect against Sugar Toxicity
Source: PLoS Genet. 2015 Feb 18;11(2):e1004995. doi: 10.1371/journal.pgen.1004995 (PMC4334898; doi:10.1371/journal.pgen.1004995)
Supplement: S3 Table — Statistical analysis was performed in R version 3.0.2, by running linear models with genotype coded as a fixed factor. “Diff DR” stands for the estimated difference in developmental rates between genotypes. Developmental rates (in units of days-1) were computed as the inverse of developmental duration to pupation. Lethality (evaluated for each developmental curve and corrected with the lethality rates for w - control measured in S5D Fig.) was accounted for by including a corresponding number of (unobserved) lethal events (individuals with a developmental rate of 0). (DOC) [file pgen.1004995.s009.doc]

| **Line** | **Genotype** | **Figure** | **Diff DR** | **p-value** |
| --- | --- | --- | --- | --- |
| 1 | *Co* vs *FASN-Ri* | **1C** | -0.0339 | 0.000*** |
| 2 | *Co* vs *ACC-Ri* | **1C** | -0.0333 | 0.000*** |
| 3 | *Co* vs *GlyS-Ri* | **1C** | -0.0091 | 0.015 * |
| 4 | *Co*  vs *FASN-Ri* | **1D** | -0.0281 | 0.000*** |
| 5 | *Co*  vs *ACC-Ri* | **1D** | -0.0305 | 0.000*** |
| 6 | *Co*  vs *GlyS-Ri* | **1D** | -0.0078 | 0.122 |
| 7 | *Co*  vs *PFK-Ri* | **S5B** | -0.0512 | 0.000*** |
| 8 | *Co*  vs *PK-Ri* | **S5B** | -0.0760 | 0.000*** |
| 9 | *Co*  vs *PFK-Ri* | **S5C** | -0.0533 | 0.000*** |
| 10 | *Co*  vs *PK-Ri* | **S5C** | -0.0728 | 0.000*** |
| 11 | *Co*  vs *glo1-Ri* | **4D** | -0.0447 | 0.000*** |
| 12 | *FASN-Ri* vs *glo1-Ri;FASN-Ri* | **4D** | -0.0722 | 0.000*** |
| 13 | *glo1-Ri* vs *glo1-Ri;FASN-Ri* | **4D** | -0.0359 | 0.000*** |
| 14 | *Co*  vs *glo1-Ri* | **4D’** | -0.0430 | 0.000*** |
| 15 | *FASN-Ri* vs *glo1-Ri;FASN-Ri* | **4D’** | -0.0569 | 0.000*** |
| 16 | *glo1-Ri* vs *glo1-Ri;FASN-Ri* | **4D’** | -0.0478 | 0.000*** |
| 17 | *Co*  vs *glo1-Ri* | **4D’’** | -0.0285 | 0.000*** |
| 18 | *FASN-Ri* vs *glo1-Ri;FASN-Ri* | **4D’’** | -0.0303 | 0.000*** |
| 19 | *glo1-Ri* vs *glo1-Ri;FASN-Ri* | **4D’’** | -0.0299 | 0.000*** |
| 20 | *Co*  vs *UAS-glo1* | **4E’** | 0.0059 | 0.002 ** |
| 21 | *FASN-Ri*  vs *FASN-Ri ;UAS-glo1* | **4E’** | -0.0138 | 0.000*** |
| 22 | *Co*  vs *UAS-glo1* | **4E’’** | 0.0124 | 0.000*** |
| 23 | *FASN-Ri*  vs *FASN-Ri ;UAS-glo1* | **4E’’** | -0.008 | 0.008 ** |

**Table S3.**
