# Supplementary material for: Fatty Acid Synthase Cooperates with Glyoxalase 1 to Protect against Sugar Toxicity
Source: PLoS Genet. 2015 Feb 18;11(2):e1004995. doi: 10.1371/journal.pgen.1004995 (PMC4334898; doi:10.1371/journal.pgen.1004995)
Supplement: S4 Table — Column 1 lists the genetic background for FASN that is either wild type (w -) hypomorph (FASN Δ24) or null (FASN Δ24-23). Column 2 and 3 indicate the usage of UAS trangenes and driver, respectively. Column 4 reports the stage of lethality (†) or the oldest developmental stage reached. L1-L3: larval stages; pp: pupal stage; ad: adult stage. Column 5 and 6 indicates the percentage of rescued animals reaching pupal and adult stages, respectively. Each test has been repeated at least 3 times. For quantification of genetic rescue, FASN mutants were combined with UAS transgenes in front of co-segregating SM5-TM6B,Tb - balancers. For UAS-FASN CG3524 rescue experiments, balanced flies were crossed together and groups of 50 Tb + L2 larvae were placed in 3 tubes, in order to determine the percentage reaching pupal rescue. For UAS-FASN CG3523 rescue experiments, balanced females were mated with homozygous males. The progeny of this cross follows a mendelian distribution, with 50% of Tb + and 50% of Tb - animals. Therefore, the percentage of rescue correspond to the number of Tb + rescued animals relative to the number of SM5-TM6B,Tb - balanced animals. (DOC) [file pgen.1004995.s010.doc]

| **background** | **UAS** | **driver** | **phenotype** | **pupal** | **adult** |
| --- | --- | --- | --- | --- | --- |
| *w^-^* | *FASN^CG3524^* | *da-gal4* | viable | NA |  |
| *w^-^* | *FASN^CG3523^* | *da-gal4* | † emb | NA |  |
| *FASN^Δ24^* | Ø | Ø | † L1 | NA |  |
| *FASN^Δ24-23^* | Ø | Ø | † L1 | NA |  |
| *FASN^Δ24-23^* | *FASN^CG3524^* | *da-gal4* | L2-3, pp | 30% | 0% |
| *FASN^Δ24^* | *FASN^CG3523^* | *da-gal4* | † emb | NA |  |
| *FASN^Δ24-23^* | *FASN^CG3523^* | *da-gal4* | † emb | NA |  |
| *FASN^Δ24^* | *FASN^CG3523^* | Ø | pp, ad | 95% | 78% |
| *FASN^Δ24-23^* | *FASN^CG3523^* | Ø | pp, ad | 54% | 18% |

**Table S4.**
