# Supplementary material for: Fatty Acid Synthase Cooperates with Glyoxalase 1 to Protect against Sugar Toxicity
Source: PLoS Genet. 2015 Feb 18;11(2):e1004995. doi: 10.1371/journal.pgen.1004995 (PMC4334898; doi:10.1371/journal.pgen.1004995)
Supplement: S5 Table — Quantities (g) of each lipid nutrient per 100ml of feeding media are indicated in the 5 top lines of the table. The various lethal stages reached by FASN Δ24 (Δ24) and FASN Δ24-23 (Δ24-23) mutant animals are indicated in the 2 bottom lines. L1-L3: larval stages; pp: pupal stage; ad: adult stage. Note that the stage of survival is variable for all rescuing media. Each test has been repeated at least 3 times. For the lipid-feeding rescue of FASN Δ24-23 mutants, groups of 30 homozygous L1 larvae were placed in 5 tubes to determine the percentage of larval, pupal and adult survival. (DOC) [file pgen.1004995.s011.doc]

| Soy lipid |  | 4g |  |  |  |  |  |  |  |
| --- | --- | --- | --- | --- | --- | --- | --- | --- | --- |
| Oil |  |  | 4g |  |  |  | 2g |  |  |
| Margarine |  |  |  | 4g |  |  |  | 2.2g |  |
| Butter |  |  |  |  | 4g |  |  |  | 2.2g |
| Egg yolk |  |  |  |  |  | 4.5g | 2.5g | 2.5g | 2.5g |
| ***Δ24 phenotype*** | **† L1** | **L1-L3, pp, ad** | **ND** | **ND** | **ND** | **ND** | **ND** | **ND** | **L1-L3, pp, ad** |
| ***Δ24-23 phenotype*** | **† L1** | **L1-L3** | **L1-L3** | **L1-L3** | **L1-L3** | **L1-L3** | **L1-L3** | **L1-L3** | **L1-L3 (73%) pp (8%), ad (19%)** |

**Table S5.**
